# Genome-wide association study in two-row spring barley landraces identifies QTL associated with plantlets root system architecture traits in well-watered and osmotic stress conditions

**DOI:** 10.3389/fpls.2023.1125672

**Published:** 2023-04-03

**Authors:** Mortaza Khodaeiaminjan, Dominic Knoch, Marie Rose Ndella Thiaw, Cintia F. Marchetti, Nikola Kořínková, Alexie Techer, Thu D. Nguyen, Jianting Chu, Valentin Bertholomey, Ingrid Doridant, Pascal Gantet, Andreas Graner, Kerstin Neumann, Véronique Bergougnoux

**Affiliations:** ^1^ Czech Advanced Technology and Research Institute, Palacký University in Olomouc, Olomouc, Czechia; ^2^ Department of Molecular Genetics, Leibniz Institute of Plant Genetics and Crop Plant Research (IPK), Gatersleben, Germany; ^3^ Unité Mixte de Recherche DIADE, Université de Montpellier, IRD, CIRAD, Montpellier, France; ^4^ Department of Breeding Research, Leibniz Institute of Plant Genetics and Crop Plant Research (IPK), Gatersleben, Germany; ^5^ Limagrain Field Seeds, Traits and Technologies, Groupe Limagrain Centre de Recherche, Chappes, France; ^6^ Department Genebank, Leibniz Institute of Plant Genetics and Crop Plant Research (IPK), Gatersleben, Germany

**Keywords:** barley landraces, osmotic stress, root system architecture, GWAS, QTL, candidate gene

## Abstract

Water availability is undoubtedly one of the most important environmental factors affecting crop production. Drought causes a gradual deprivation of water in the soil from top to deep layers and can occur at diverse stages of plant development. Roots are the first organs that perceive water deficit in soil and their adaptive development contributes to drought adaptation. Domestication has contributed to a bottleneck in genetic diversity. Wild species or landraces represent a pool of genetic diversity that has not been exploited yet in breeding program. In this study, we used a collection of 230 two-row spring barley landraces to detect phenotypic variation in root system plasticity in response to drought and to identify new quantitative trait loci (QTL) involved in root system architecture under diverse growth conditions. For this purpose, young seedlings grown for 21 days in pouches under control and osmotic-stress conditions were phenotyped and genotyped using the barley 50k iSelect SNP array, and genome-wide association studies (GWAS) were conducted using three different GWAS methods (MLM GAPIT, FarmCPU, and BLINK) to detect genotype/phenotype associations. In total, 276 significant marker-trait associations (MTAs; *p*-value (FDR)< 0.05) were identified for root (14 and 12 traits under osmotic-stress and control conditions, respectively) and for three shoot traits under both conditions. In total, 52 QTL (multi-trait or identified by at least two different GWAS approaches) were investigated to identify genes representing promising candidates with a role in root development and adaptation to drought stress.

## Introduction

Barley (*Hordeum vulgare* L.) is one of the oldest domesticated crops, cultivated as early as 8000 BC in Persia as human food and livestock feed (Sullivan et al., 2013). Nowadays, barley is the fourth most important cereal worldwide. Its early maturity, diploidy with low chromosome number (n=7), self-pollination and its adaptative plasticity make barley an ideal model crop (Pourkheirandish and Komatsuda, 2007; Saisho and Takeda, 2011). Deliberate or natural selection, as well as spontaneous mutations have contributed to the rich genetic variability of barley landraces, providing the base material for modern genetic studies (Kumar et al., 2020a).

The rapidly growing human population increases the need for food production. Reduction in water availability have a negative impact on worldwide barley production (Jamieson et al., 1995). Drought can occur at any growth stage of the plants’ life cycle in different environments. Depending on the adaptative mechanism, some genotypes can tolerate drought at a particular growth stage but might be sensitive at other stages. The seedling stage is a very critical period. In regions with low precipitation, where optimal water availability can be observed at the time of sowing, drought stress may occur shortly after germination (Sallam et al., 2019), severely affecting barley development in the juvenile stages, and consequently reducing yields (Wehner et al., 2015).

Roots are the first plant organs that perceive water deficit in the drying soil (Ksouri et al., 2016). The crucial role of the root system in water acquisition as well as in adaptation and tolerance to water‐deficit have been reported previously (Ehdaie et al., 2012; Lynch et al., 2014; Palta and Yang, 2014; Paez-Garcia et al., 2015; Geng et al., 2018). Root system architecture (RSA) is defined as the number and geometric arrangement of individual embryonic roots (primary and seminal) and postembryonic roots (lateral or adventitious roots) in the three-dimensional soil space (Steffens and Rasmussen, 2016; Del Bianco and Kepinski, 2018). RSA determines the ability of a plant to explore and to exploit unevenly distributed soil resources. In maize, deeper, and thinner root systems are morphologically more favourable adaptation traits to drought than shallow and thick root systems (Lynch, 2015). Longer primary roots and elongated root hairs constitute important traits in cereals adaptation to drought stress (Wasson et al., 2012; Lynch, 2013).

Root system characteristics are both genetically and environmentally determined (Lynch and Brown, 2012). In barley, RSA is a complex multi-traits phenotype, genetically controlled by numerous genes. Different genes can contribute to RSA formation at different stages of plant growth in response to drought stress, hence the identification of the genetic mechanisms underlying the RSA in barley seedlings is crucial for breeding programs. Despite the importance of RSA in water deficit responses, roots are generally less frequently analysed than aboveground organs because of the difficulties of observation. Thereupon, the role of roots in barley water deficit response and tolerance, and its genetic bases remain elusive. Genome‐wide association study (GWAS) is a method widely used in crops to dissect the genetic bases of highly complex quantitative traits (Fang et al., 2017; Oyiga et al., 2018; Oyiga et al., 2019). Recently, Jia et al. (2019) reported three major quantitative trait loci (QTL) in barley controlling different root system parameters in normal growth condition. Eleven QTL involved in the nodal root variation in response to water deficit have been reported for barley plants grown in field (Oyiga et al., 2020). A first attempt was made recently to study RSA seedling traits in barley by the paper roll technique in hydroponic solution where osmotic stress was induced by polyethylene glycol (PEG) (Abdel-Ghani et al., 2019).

In the present study, a collection of 230 two-row spring barley landraces originated from three geographical regions (Europe, Asia, and Africa) (Pasam et al., 2014) was analysed to dissect the genetic bases of RSA in response to osmotic stress at the seedling stage. For this purpose, we performed GWAS based on the 50k iSelect SNP genotyping data (Bayer et al., 2017) and root architecture parameters determined for barley plantlets grown in germination pouches under control and osmotic stress conditions. Three computational approaches (MLM GAPIT, FarmCPU, BLINK) were used and the most promising associations, linked to multiple traits or detected by at least two GWAS approaches, were further investigated to determine candidate genes with a role in root development and adaptation to drought.

## Materials and methods

### Plant material and growth conditions

A subset of the spring barley landrace collection maintained at the Federal ex-situ GenBank for Agricultural and Horticultural Crop Species at the Leibniz Institute of Plant Genetics and Crop Plant Research (IPK) Gatersleben, Germany, was used for genetic dissection of the RSA and shoot traits under osmotic stress and control conditions (**Supplementary Table S1**). The collection was established by several rounds of single seed descend and harbours rich diversity (Pasam et al., 2014; Wabila et al., 2019). The sub-panel used in this study, comprises 230 two-row spring barley landraces covering three geographical regions (Europe, Asia, and Africa) and originating from 31 countries. Of them, 190 develop hulled-grains and 40 naked-grains.

Grains were sterilized in 70% ethanol for 30 seconds and washed three times with sterile distilled water. Subsequently, seeds were soaked into 5% sodium hypochlorite for 5 minutes with regular shaking and intensively rinsed with sterile ddH_2_O. Sterile CYG™ germination pouches (16.5 cm by 18 cm, Mega international, USA) were moistened with 50 ml sterile ddH_2_O. Grains from the same genotype were sown in two groups (left and right; three seeds per group) in perforations of the germination paper with the embryos pointing downward. Pouches were placed in dark plastic boxes and covered with aluminium foil. The full system was kept in a cold room at 4°C for three days to ensure homogeneous breaking of dormancy (stratification). Following stratification, the full system was transferred to a phytotron with controlled conditions (photoperiod: 12/12h; 13°C (night)/16°C (day); light intensity: 270 μmol photons.m^-2^.s^-1^; 60% relative humidity) for germination. Four days after germination, only two homogeneously germinated seedlings were kept per pouch (left and right). For control conditions, water was exchanged by ½ strength modified Hoagland nutrient solution (Vlamis and Williams, 1962) supplemented with 2 ml/l of fungicide (Previcur Energy, Bayer Garden); after 7 days, the solution was refreshed and the plants were grown for another 7 days. For osmotic stress conditions, water was exchanged by 15% (w/v) polyethylene glycol 8000 (PEG_8000_) in ½ -strength modified Hoagland nutrient solution containing 2 ml/l fungicide. After 7 days, the solution was refreshed by ½ -strength modified Hoagland nutrient solution containing 2 ml/l fungicide and 25% (w/v) PEG_8000_; seedlings were further grown for 7 days. For all the duration of the experiment (14 days after germination: 14DAG), roots were prevented from light exposure and the growing system was kept in a phytotron under controlled conditions (photoperiod: 12/12h; 13°C (night)/16°C (day); light intensity: 270 μmol photons m-2 s-1; 60% relative humidity). A schematic representation of the experimental design can be found in **Supplementary Figure S1**. Control and stress conditions were applied in parallel and analysed at the same time. The full experiment was conducted twice, providing two independent experiments. In total, each genotype in both osmotic stress and control conditions were represented by four biological replicates.

### Root system and biomass phenotyping

At the end of the growing period (21 days; 14 days-old plantlets), the relative water content (RWC) was determined for each plant as described by Barrs and Weatherley (1962). The root system of each plantlet was scanned using the ImageScanner III LabScan 6.0 with a 600-dpi resolution and saved as tiff-formatted picture. Shoot and root fresh weight (SFW and RFW), fresh root/shoot ratio (RSR), and total biomass (TotalBio), were determined for each plant. Furthermore, roots and shoots were dried at 70°C for 48h, and shoot and root dry weights (SDW and RDW) were measured. Images of the roots were analysed using an ImageJ macro to convert the root system in white pixels on a black pixel background. Those images were analysed with GiA Roots software (Galkovskyi et al., 2012) that can extract up to 20 traits characterizing the root system (**Supplementary Figure S1**). In total, 27 traits were obtained for both osmotic stress and control conditions (**Table 1**).

**Table 1:** Description of 27 root and shoot traits analyzed.

Trait	Abbreviation	Description	Unit	GWAS
*Max. Number of roots*	Max.NR	After sorting the number of roots crossing a horizontal line from smallest to largest, the maximum number is considered to be the 84th-percentile value	n	Yes
*Median Number of roots*	Med.NR	The result of a vertical line sweep in which the number of roots that crossed a horizontal line was estimated, then the median of all values for the extent of the network was calculated	n	No
*Network Bushiness*	Net.Bush	The ratio of the maximum to the median number of roots	n/n	No
*Convex area*	Conv.A	The area of the convex hull that encompasses the root	cm ^2^	Yes
*Network depth*	Net.Dep	The number of pixels in the vertical direction from the upper-most network pixel to the lower-most network pixel	cm	Yes
*Network Length Distribution*	Net.Len.Dis	The lower 2/3 of the network is defined based on the network depth	n/n	Yes
*Major Ellipse Axis*	Maj.A	The length of the major axis of the best fitting ellipse to the network	cm	No
*Network Width*	Net.Width	The number of pixels in the horizontal direction from the left-most network pixel to the right-most network pixel. Only pixels lying in the same row are considered	cm	Yes
*Average root width (diameter)*	Av.R.Width	The mean value of the root width estimation computed for all pixels of the medial axis of the entire root system. This trait corresponds to diameter of a root	cm	No
*Minor Ellips Axis*	Min.El.A	The length of the minor axis of the best fitting ellipse to the network	cm	No
*Network area*	Net.A	The number of network pixels in the image	cm^2^	Yes
*Perimeter*	Perim	The total number of network pixels connected to a background pixel	cm	No
*No. of connected components*	N.Con.Com	The number of connected groups of network pixels in the image after image pre-processing	n	No
*Network solidity*	Net.Solidity	The total network area divided by the network convex area	cm^2^/cm^2^	Yes
*Specific root length*	SRL	Total network length divided by network volume	cm/cm^3^	No
*Network Surface Area*	Net.Surf	The sum of the local surface area at each pixel of the network skeleton, as approximated by a tubular shape whose radius is estimated from the image	cm^2^	No
*Network length*	Net.Len	The total number of pixels in the network skeleton.	cm	Yes
*Network volume*	Net.vol	The sum of the local volume at each pixel of the network skeleton, as approximated by a tubular shape whose radius is estimated from the image.	cm 3	No
*Network width to depth ratio*	Net.Wid/Dep	The value of network width divided by the value of network depth.	cm/cm	Yes
*Ellipse Axes Ratio*	El.A.R	The ratio between major and minor axis of ellipse created around the roots	cm/cm	No
*Relative water content*	RWC	[(Sample fresh weight - sample dry weight)/(Sample turgid weight – Sample dry weight)] x 100	%	Yes
*Shoot fresh weight*	SFW	Fresh weight of shoot for each individual plant	mg	Yes
*Shoot dry weight*	SDW	The weight of the shoot part dried at 70°C for 48h in each individual plant	mg	Yes
*Root fresh weight*	RFW	Fresh weight of root for each individual plant	mg	Yes
*Root dry weight*	RDW	The weight of the root part dried at 70°C for 48h in each individual plant	mg	Yes
*Total Biomass*	Total.Bio	Total fresh weight of root and shoot for each individual plant	mg	Yes
*Root/Shoot Ratio*	RSR	Root fresh weight to shoot fresh weight ratio	mg	Yes

The traits considered for GWAS are indicated.

### Data analysis

Phenotypic analysis was performed in R software (R Core Team, 2021). The outlier test was performed according to Tukey’s method (Anscombe and Tukey, 1963). Outliers, within and across the experiments were removed. Genotypes with >20% missing phenotypic data were not considered for further analysis. Heritability (H^2^) of each trait was estimated by equations (1 & 2) using the ‘ASReml’ R package (Gilmour et al., 1995). Repeatability values (R) were estimated by the equations (2 & 3). Best linear unbiased estimates (BLUEs) were calculated based on the linear model described by the equation (4) considering the experiments and genotype X experiment (GxE) interactions for each trait applying the Restricted Maximum Likelihood (REML) method. $\sigma_{G}^{2}$ , $\sigma_{GxE}^{2}$ , and $\sigma_{\text{e}}^{2}\;$ denote the variance components of the genotype, the genotype x experiment interaction, and the residuals, respectively. n_0_ is the number of experiments, and $n_{R}$ the number of biological replicates.


(1) $$H^{2}=\;\frac{\sigma_{G}^{2}}{\sigma_{G}^{2}\;+\;\frac{\sigma_{GxE}^{2}}{n_{0}}\;+\;\frac{\sigma_{e}^{2}}{n_{0\;}x\;n_{R}}}$$

(2) Y=G+E+GxE+e

(3) $$R=\;\frac{\sigma_{G}^{2}}{\sigma_{G}^{2}\;+\;\frac{\sigma_{e}^{2}}{n_{R\;}}}$$

(4) Y=G+GxE+e

### Genotype data and population structure

All 230 lines were genotyped using the Barley 50k Illumina Infinium iSelect SNP array (Bayer et al., 2017). From the total number of 44,040 SNP markers on the array, 38,349 polymorphic markers were scored. Marker positions on the barley Morex v3 reference genome version (Mascher et al., 2021) were obtained from BARLEX (https://apex.ipk-gatersleben.de/apex/f?p=284:63; Colmsee et al., 2015). SNP markers were filtered for missing data (>20% for the lines and >10% for the markers) and minor allele frequency (MAF > 0.05) resulting in a set of 32,286 markers for subsequent analyses. Missing genotype calls were imputed using BEAGLE v5 (Browning and Browning, 2007; Browning et al., 2018). The data have also been deposited at the European Variation Archive (EVA; https://www.ebi.ac.uk/eva/?eva-study=PRJEB59438). Due to discrepancies in marker positions between BARLEX and the Germinate Barley SNP Platform (https://ics.hutton.ac.uk/50k/) that could not be resolved, 732 markers could not be uploaded to EVA and are given as supplemental genomic dataset (**Supplementary SNP Dataset**).

Population structure was estimated by a principal component analysis (PCA) using singular value decomposition. The analysis was performed on the centered genotype data using the PCA function of the ‘pcaMethods’ R package (Stacklies et al., 2007). The first ten principal components were calculated. A phylogenetic tree was built based on the neighbour-joining algorithm (Saitou and Nei, 1987) using the SNP marker data and the nj function of the ‘ape’ R package (Paradis et al., 2004; Paradis and Schliep, 2019). Bootstrapping with 100 bootstrap replicates was performed using the boot.phylo function. In addition, the population structure was analysed using the STRUCTURE program version 2.3.4 (Pritchard et al., 2000) using the ‘admixture’ model. Population clustering for K= 1 to 12 was analysed with a burn-in period of 10.000 and 50.000 MCMC replications, each with 10 iterations per K. The optimal number of clusters (K= 8) was determined by the ΔK method introduced by (Evanno et al., 2005).

### Genome-wide association study (GWAS)

In this study we used 32,286 high-quality SNP markers (MAF ≥ 0.05) to perform genome-wide association analyses (GWAS). Association analyses were performed in R version 4.0.2 (R Core Team, 2021) using BLUEs of the phenotypic traits. To identify QTL, we employed not only a classical single locus mixed linear model (MLM) correcting for population structure and kinship (Q+K) using the Genomic Association and Prediction Integrated Tool (GAPIT; Lipka et al., 2012; Wang and Zhang, 2020), but also two state-of-the-art multivariate GWAS methods: Fixed and random model Circulating Probability Unification (FarmCPU; Liu et al., 2016) and Bayesian-information and Linkage-disequilibrium Iteratively Nested Keyway (BLINK; Huang et al., 2019).

In GAPIT, we accounted for population structure (Q) through principal components (PCs) and for relationships among individuals through a kinship (K) matrix (VanRaden, 2008), both calculated based on the marker data. FarmCPU and BLINK were run with default settings with two exceptions: the maxLoop parameter was increased from 10 to 100 for both methods and for FarmCPU, the optimal threshold for p-value selection of the model in the first iteration was set to p.threshold=0.0000015 (a Bonferoni-corrected threshold with 0.05/number of markers, rounded to the seventh decimal) for all traits. To deal with the effects of population structure, the number of PC covariates included in the different models was optimized. Indeed, the number of PC covariates included in the GWAS model can have a substantial impact on the results, as it depends heavily on the genetic architecture of the trait of interest (Malik et al., 2019; Meyer et al., 2021). Consequently, all GWAS methods were performed with different PC numbers ranging from 0 to 10, with the optimal PC number for each trait being chosen based on the quantile-quantile (QQ) plots. This strategy has commonly been used to determine whether a model effectively controls false positives and false negatives (Stich et al., 2008; Stich and Melchinger, 2009; Riedelsheimer et al., 2012; Würschum et al., 2012; Kristensen et al., 2018). Subsequently, p-values of marker-trait-associations (MTAs) were adjusted for multiple comparisons using FDR (Benjamini and Hochberg, 1995). MTAs with adjusted p-value (FDR)< 0.05 were considered as statistically significant and< 0.1 as potentially interesting. Both were kept for further analyses. GWAS results were visualized by Manhattan plots generated using the ‘rMVP’ package (Yin et al., 2021). The phenotypic variance explained (PVE%) by a significant marker was estimated in R. The sum of squares (SS) and residuals (e) were extracted from the ANOVA fitted with a linear model incorporating the phenotypic values and all markers with p-value_FDR_ < 0.1 in decreasing order. MTAs identified at least with two GWAS tools together with multi-trait MTAs were mapped on the seven barley chromosomes using MapChart 2.32 Windows (Voorrips, 2002).

### Linkage disequilibrium (LD) analysis and nomination of candidate genes

Pairwise linkage disequilibrium (LD) was analysed for each chromosome in R using the ‘LDheatmap’ package (Shin et al., 2006). The decay was calculated in R for all chromosomes separately (Hill and Weir, 1988; Remington et al., 2001; Marroni et al., 2011).

For comparing associations detected with different methods, MTAs were grouped in LD blocks if directly neighbouring markers displayed strong LD (r2 > 0.5). All genes within such a specific LD block were considered for candidate gene identification, whereby the search intervals were extended to the left and right neighbouring marker, respectively. For significant MTAs outside of LD blocks, the region defined by the flanking markers were searched for candidate genes.

Only genes with high confidence annotation, as determined from the barley annotation, were considered. Their corresponding protein sequence were retrieved from the Morex v3 reference genome and subjected to functional annotation The widely used gene ontology (GO) comprises more than 34,000 terms organized in 3 categories: “Biological process”, “Molecular Function” and “Cellular component”. This rich annotation can lead to a strong redundancy. In opposite, Mapman, specifically developed for plants, assigns genes to as few functional categories as possible without losing information. Currently, MapMan ontology covers 27 functional top-categories (Klie and Nikoloski, 2012; Schwacke et al., 2019). Therefore, the functional annotation of the candidate genes was done using the MapMan BIN ontology in Mercator (Lohse et al., 2014). For both control and stress conditions, the number of genes entering a specific BIN category was retrieved and plotted as histogram representation.

## Results

A collection of 230 two-row spring barley landraces was investigated to study the genetic basis of RSA of 14DAG old-seedlings grown under control and osmotic-stress conditions. Using 20 barley genotypes representative the diversity of the collection, a pilot experiment was conducted with different percentages of PEG_8000_ to optimize the osmotic stress condition based on RWC and total biomass. Significant reduction in RWC and TotalBio were observed using 15% and 25% of PEG for the first and second week, respectively (data not provided). All 230 barley landraces were grown and phenotyped in CYG™ germination pouches and a total number of 16 root and shoot traits were analysed for plants grown both in control conditions and osmotic stress. RWC (*p*-value = 2.26E-46) and TotalBio (*p*-value = 1.09E-83) were significantly reduced under osmotic stress. A total number of 32,286 filtered SNP markers from the 50k iSelect SNP array (Bayer et al., 2017) with marker positions based on Morex v3 reference genome version (Mascher et al., 2021) were used in combination with the phenotypic traits scored under both conditions for GWAS, followed by the identification of genes potentially associated with RSA and response to drought stress in barley.

### Phenotypic data analysis

In total, 10 genotypes were omitted in both stress and control conditions due to exceeding the missing data threshold (**Supplementary Table S1**). Heritability, repeatability, and BLUEs were estimated for each trait in both conditions. Heritability values (H^2^) ranged from 0.00 for Net.Bush to 0.77 for Net.Area and RDW in control conditions. Under osmotic stress conditions, the lowest and highest H^2^ values were obtained for specific root length (0.07) and RDW (0.73), suggesting that the RDW was under strong genetic control in both stress and control condition (**Supplementary Table S2**). Under control conditions, the Net.Bush (0.00) and RSR (0.036) in the first experiment, together with TotalBio in the second experiment showed the lowest repeatability, while the lowest repeatability under osmotic stress was observed for Net.Bush in experiment one and TotalBioin experiment two. Net.Dep showed the highest repeatability value in both experiments under control (0.718) and osmotic stress (0.737) (**Supplementary Table S3**). The traits Net.Bush and specific root length were not further considered.

BLUEs were calculated within and across the experiments for each of the 27 root and shoot traits for 220 genotypes in both control (**Supplementary Table S4**) and osmotic stress (**Supplementary Table S5**). BLUEs for each trait were used for statistical analysis and GWAS. For most traits, the landrace collection showed a high level of phenotypic diversity. The coefficients of variation ranged from 1.69 for RWC to 28.69 for Net.Con.A under control conditions. Under osmotic stress, the lowest coefficient of variation (2.45) was observed for RWC but increased compared to control, and the highest value (59.18) was observed for the number of connected components (**Supplementary Table S6**). We evaluated how the osmotic stress treatment influenced phenotypic trait values. Under osmotic stress, the Net.Len.Dis (9.64%), Net.Solid (5.30%) and specific root length (91.49%) increased while all other traits showed a reduction from 0.6% for RDW to 60.5% for Net.Area (**Supplementary Table S6**). Further, the correlation for each trait in both conditions was tested. The lowest treatment correlation (0.065) was observed for specific root length and the highest value (0.56) was observed for SDW (**Supplementary Table S6**), implying a strong effect of osmotic stress on genotype ranking.

We compared the phenotypic results of the two caryopsis types present in the landrace collection (hulled vs naked) and observed a great difference on the root and shoot traits under osmotic stress and control conditions within the two groups. Interestingly, the SDW was significantly higher in naked types under both conditions while no significant differences in SFW under both osmotic stress and control conditions (**Supplementary Table S7**) were observed. In general, most traits were significantly higher in hulled types or showed no significant differences in control condition (**Supplementary Table S7**). However, Net.Bushines under control and, Net.Solidity under osmotic stress and SDW under both conditions were higher in the naked group in both conditions. Specific root length was higher in naked barley in control conditions while under osmotic stress, the hulled genotypes had the higher Specific root length. Average root width in contrast, was only higher in the naked group under osmotic stress conditions. Considering the correlation between the traits (**Supplementary Tables S8A, B**), that some of the parameters obtained from image analysis are related to similar root architecture parameters and taking heritability and repeatability values of different traits into consideration, 9 non-redundant, heritable, and repeatable RSA traits obtained from images analysis together with seven traits manually determined (**Table 1**) were selected, for GWAS. The network area (Net.Area) constitutes a proxy of the total root biomass, the network length (Net.Len) measures the total root length and the maximum number of roots (Max.NR) estimates the branching degree of the RSA. Deepness of the RSA is measured with the network depth (Net.Dep), network length distribution (Net.Len.Dis) estimates the root biomass in depth. The width is measured by the network width (Net.Width), the network width to depth ratio (Net.Width/Depth Ratio) is giving a description of the general shape of the RSA and root density. The soil portion explored by the root system is estimated by the convex area (Net.Con.A). and the root density by network solidity (Net.Solidity).

### Population structure and LD analysis

**Figure 1:**
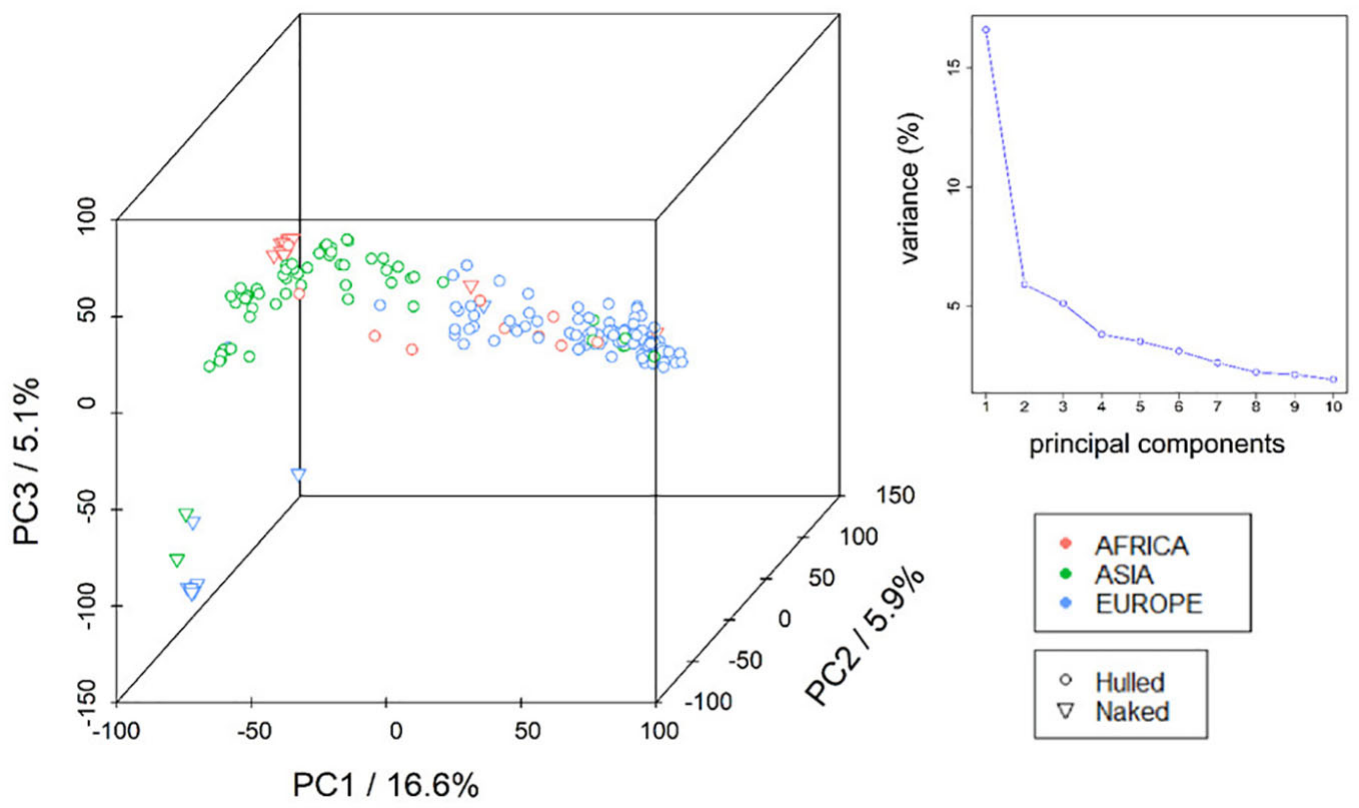
Visualisation of population structure (PCA). A principal component analysis (PCA) was performed on the panel of 206 two-rowed Barley landraces using 32,286 SNP markers to visualize population structure. Proportions of explained variance of principal components (PCs) 1, 2 and 3 are indicated on the axes. Different colours correspond to the geographic origin (continents: red = Africa, green = Asia, blue= Europe) of the lines according to collection information. Symbols indicate grain features with circles and triangles representing hulled and naked lines, respectively.

To deal with the population structure in GWAS, a principal component analysis (PCA) was performed on the panel of 206 barley landraces (fourteen genotypes were omitted due to low quality of genotypic data) using 32,286 SNP markers distributed across the seven barley chromosomes (**Supplementary Figure S2**). The first ten principal components explain a cumulative variance of approx. 47% (**Supplementary Table S9**). The PCA indicates the existence of population structure in the panel of barley landraces (**Figure 1**), coinciding with the geographical origin of the genotypes. This finding is supported by the calculation of a neighbour joining (NJ) tree (**Supplementary Figure S3**). European landraces clustered together, while the lines from Asia formed two separate groups. Moreover, the Ethiopian landraces which are mainly naked types, formed a separate group. One smaller subgroup consisted of landraces from Europe and Asia. In the STRUCTURE analysis the mean Ln probability L(K), ΔK, and population clustering for K= 3 to 8 are shown in **Figure S3**. (**Supplementary Figure S4**) we identified eight Q-groups. Pairwise marker LD-matrices (r2) were calculated for each chromosome and LD-decay analysed for each chromosome separately shows an overall sharp LD-decay for all seven chromosomes with half-maximum-LD between 712 kb and 1.2 Mb (**Supplementary Figure S5**).

### Genome-wide association analyses

For GWAS, we focused on the16 selected traits. Three different methods were employed for GWAS: GAPIT with a univariate mixed linear model (MLM) approach, FarmCPU and BLINK using multi-locus models. In addition, we calculated for all traits the ratios between osmotic stress and control conditions and subjected them to the same GWAS pipeline. The ratios provide information about phenotypic plasticity and how well lines are able to maintain their root system architecture characteristics under stress conditions.

In summary, 276 significant marker-traits associations (MTAs; p-value_FDR_< 0.05) and 70 potentially interesting associations were identified (p-value_FDR_ 0.1) across all three methods and traits, whereby individual MTAs explained on average 4.4% and for individual phenotypic traits up to 48.95% of phenotypic variance (PVE) (**Supplementary Table S10**). These associations were distributed across the seven barley chromosomes (n 1H = 34, n 2H = 69, n 3H = 42, n 4H = 75, n 5H = 44, n 6H = 40, and n 7H = 42). Notably, just two associations (‘BOPA1_1582-63’ on chromosome 5H for root/shoot ratio and ‘SCRI_RS_192761’ on chromosome 3H for shoot dry weight) were shared between the same traits scored under control and osmotic stress conditions. In addition, three common loci (‘JHI-Hv50k-2016-86571’, ‘LD block 2H - 774’, and ‘LD block 4H - 487’) were detected under both conditions but associated with different traits.

**Figure 2:**
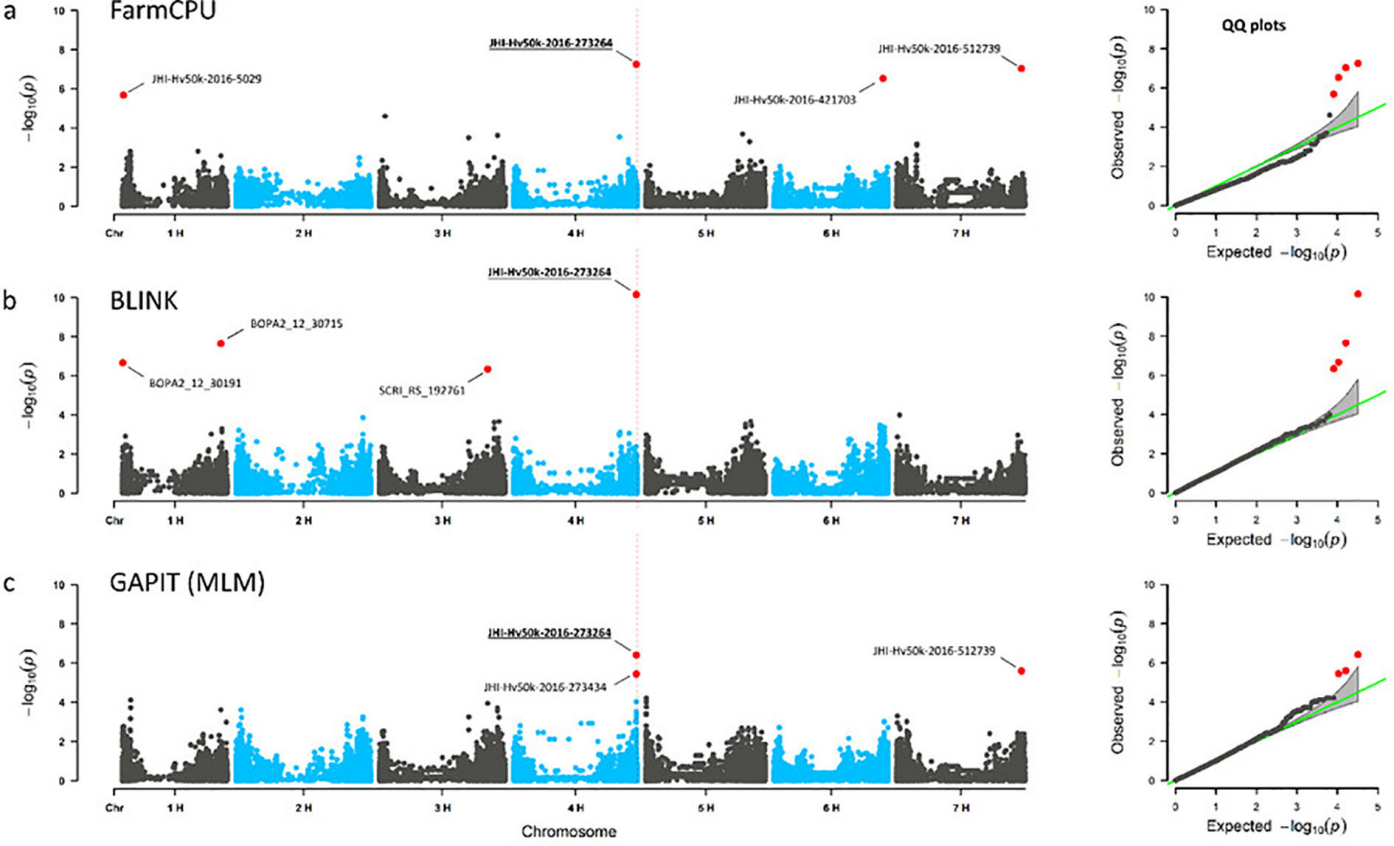
Consensus QTL for shoot dry weight under stress conditions (SDW-PEG). Genome-wide marker-trait associations for shoot dry weight under osmotic stress conditions (SDW-PEG). Manhattan plots (left) and quantile-quantile plots (right), **(A)** for FarmCPU, **(B)** BLINK and **(C)** GAPIT using MLM. GWAS were performed on BLUEs. Significant marker-trait-associations (MTAs) are shown in red colour with marker-IDs. MTAs with *p*-value _(FDR)_< 0.05 indicated by red dots. SNP marker JHI-Hv50k-2016-273264 on chromosome 4H was detected in all three GWAS methods.

Using GAPIT with an MLM model and optimized PC numbers, a total of 14 significant MTAs (13 markers/LD blocks) was obtained for three traits under osmotic stress conditions and none under the control conditions (**Supplementary Table S10**). By BLINK, 26 significant MTAs (24 markers/LD blocks) were identified for 10 of the phenotypic traits in control condition and 59 significant MTAs (53 markers/LD blocks) for 15 traits under osmotic stress (**Table S10**). Using FarmCPU, 65 significant MTAs (61 markers/LD blocks) were identified for all 16 traits under osmotic stress condition, while under control condition 62 significant MTAs (53 markers/LD blocks) were identified for 15 of the phenotypic traits (**Table S10**). To find common associations between the three approaches, we searched for significant MTAs passing the FDR threshold< 0.05 in one of the models and passing the FDR threshold< 0.1 in at least one another model (**Table 2**). Finally, 26 MTAs/LD blocks were identified both by BLINK and FarmCPU, and one MTA by GAPIT and FarmCPU for the same traits. Three MTAs, ‘JHI-Hv50k-2016-273264’ on chromosome 4H associated with SDW under osmotic stress conditions (SDW-PEG), ‘JHI-Hv50k-2016-153756’ on chromosome 3H associated with the RWC under osmotic stress conditions (RWC-PEG) (**Figure 2**), and ‘LD block 4H - 345’ on chromosome 4H associated with the RSR-stress/control-ratio were identified by all three GWAS methods (**Supplementary Table S10**). These 29 MTAs related to 27 genomic loci were further considered for the identification of genes potentially involved in root growth of barley under osmotic stress and control condition.

**Table 2:** List of markers-trait associations identified for osmotic stress and control condition by at least two GWAS methods.

Trait	Condition	Marker	Chr	Position	Method	*p*-value (FDR)	PVE%	LD block
Max.NR-PEG	Osmotic stress	BOPA2_12_30275	2H	475,258,998	BLINK	1.30E-03	5.63	LD Block 2H - 370
					FarmCPU	2.30E-02	9.59	
Net.Area	Control	JHI-Hv50k-2016-464245	7H	55,410,960	BLINK	4.69E-03	7.06	LD Block 7H - 243
					FarmCPU	2.14E-02	5.34	
Net.Area-PEG	Osmotic stress	JHI-Hv50k-2016-93675	2H	402,933,731	BLINK	2.46E-02	4.73	LD Block 2H - 354
					FarmCPU	2.18E-03	2.69	
		JHI-Hv50k-2016-187836	3H	477,631,357	BLINK	3.85E-04	10.1	-
					FarmCPU	8.04E-07	8.55	
Net.Con.A-PEG	Osmotic stress	BOPA2_12_10166	1H	437,092,173	BLINK	4.00E-05	8.04	-
					FarmCPU	4.31E-07	7.56	
		JHI-Hv50k-2016-228324	4H	6,499,257	BLINK	1.66E-02	2.15	LD Block 4H - 43
				6,500,571	FarmCPU	2.53E-05	5.33	
Net.Dep-PEG	Osmotic stress	JHI-Hv50k-2016-77039	2H	39,484,827	BLINK	1.44E-06	8.23	-
					FarmCPU	6.21E-03	7.07	
		JHI-Hv50k-2016-250777	4H	499,577,241	BLINK	4.05E-02	0.87	-
					FarmCPU	3.11E-04	3.67	
Net.Len	Control	JHI-Hv50k-2016-93361	2H	388,503,654	BLINK	2.56E-02	3.25	-
					FarmCPU	4.07E-02	9.02	
Net.Len.Dis-PEG	Osmotic stress	JHI-Hv50k-2016-382108	6H	32,581,204	BLINK	2.52E-02	3.24	LD Block 6H - 159
					FarmCPU	2.51E-02	8.94	
Net.Len-PEG	Osmotic stress	SCRI_RS_154135	2H	647,527,751	BLINK	9.47E-03	5.48	LD Block 2H - 866
					FarmCPU	9.22E-02	5.26	
		JHI-Hv50k-2016-187836	3H	477,631,357	BLINK	5.14E-04	8.24	-
					FarmCPU	4.35E-03	9.4	
Net.Solid-PEG	Osmotic stress	JHI-Hv50k-2016-260522	4H	566,353,428	BLINK	2.46E-03	11.38	LD Block 4H - 487
Net.Width	Control	JHI-Hv50k-2016-317067	5H	489,074,858	BLINK	6.61E-02	9.67	-
					FarmCPU	1.01E-04	9.35	
RDW	Control	JHI-Hv50k-2016-413564	6H	502,948,428	BLINK	8.00E-04	1.88	LD Block 6H – 488
					FarmCPU	1.73E-02	2.79	
RDW-PEG	Osmotic stress	JHI-Hv50k-2016-277549	5H	2,137,151	BLINK	1.04E-03	1.63	LD Block 5H - 13
		JHI-Hv50k-2016-277552		2,137,666	FarmCPU	2.65E-04	1.79	
RFW	Control	JHI-Hv50k-2016-311308	5H	464,498,212	BLINK	1.62E-02	3.53	LD Block 5H - 449
					FarmCPU	6.03E-03	3.48	
RFW-PEG	Osmotic stress	BOPA2_12_10554	6H	14,517,119	BLINK	4.12E-03	1	–
					FarmCPU	3.48E-03	1.22	
RSR-PEG	Osmotic stress	JHI-Hv50k-2016-247715	4H	466,110,916	BLINK	2.25E-03	22.69	–
					FarmCPU	3.85E-03	4.45	
RSR-PEG/Control-ratio	Ratio	JHI-Hv50k-2016-250905	4H	500,121,703	GAPIT	9.89E-02	1.83	LD Block 4H - 345
		JHI-Hv50k-2016-250920		500,270,838	BLINK	3.30E-04	2.06	
					FarmCPU	3.58E-04	2.13	
					GAPIT	9.89E-02	0.27	
		JHI-Hv50k-2016-317209	5H	489,458,348	BLINK	4.35E-03	4.06	–
					FarmCPU	3.61E-02	1.35	
RWC-PEG	Osmotic stress	JHI-Hv50k-2016-153756	3H	8,583,074	BLINK	1.44E-07	8.8	–
					FarmCPU	3.56E-03	8.57	
					GAPIT	2.73E-02	7.9	
SDW	Control	JHI-Hv50k-2016-518088	7H	626,758,933	BLINK	7.49E-02	3.48	–
					FarmCPU	9.79E-02	1.37	
SDW-PEG	Osmotic stress	JHI-Hv50k-2016-273264	4H	604,203,108	BLINK	2.24E-06	0.58	–
					FarmCPU	1.51E-03	0.56	
					GAPIT	1.25E-02	0.52	
		JHI-Hv50k-2016-512739	7H	615,801,734	FarmCPU	1.51E-03	14.71	–
					GAPIT	3.89E-02	13.73	
SFW-PEG	Osmotic stress	BOPA2_12_30191	1H	485,117,907	BLINK	5.64E-02	15.01	–
					FarmCPU	9.53E-02	14.48	
		JHI-Hv50k-2016-273264	4H	604,203,108	BLINK	2.09E-07	1.34	–
					FarmCPU	2.68E-04	1.3	
		JHI-Hv50k-2016-417034	6H	523,026,467	BLINK	1.85E-02	0.53	LD Block 6H - 552
					FarmCPU	1.32E-02	0.33	
TotalBio-PEG	Osmotic stress	JHI-Hv50k-2016-82606	2H	70,465,843	BLINK	2.30E-03	4.54	LD Block 2H - 247

**Figure 3:**
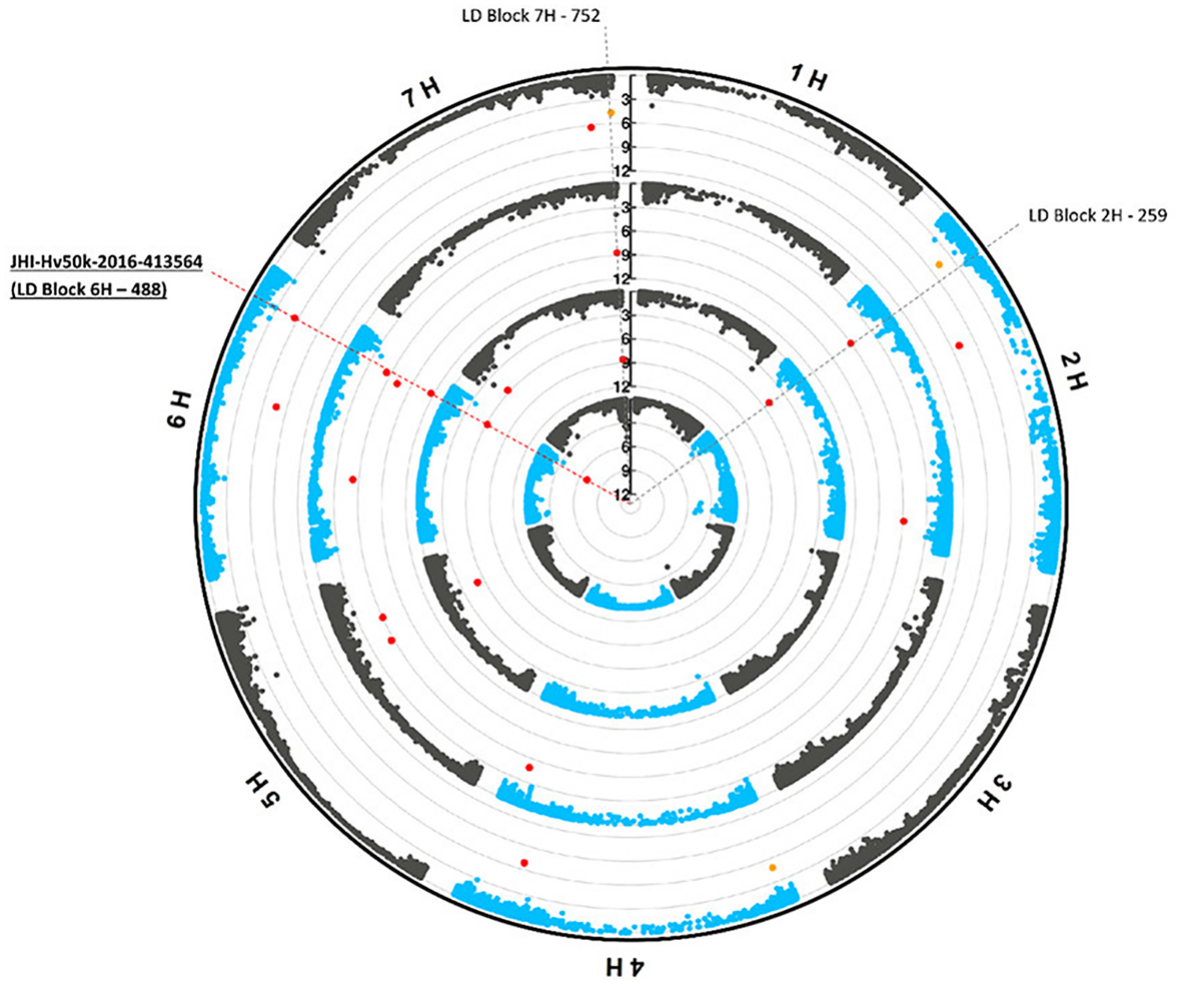
Multi-trait marker (JHI-Hv50k-2016-413564) on chromosome 6H. Circular Manhattan plots (FarmCPU) for the phenotypic traits root dry weight (RDW), root fresh weight (RFW), Net.Area, and TotalBio under control conditions are shown from the inner to outer circles, respectively. Significant marker-trait associations (*p*-value _(FDR)_< 0.05) and potentially interesting associations (*p*-value _(FDR)_< 0.1) are indicated by red and orange dots, respectively. The SNP markers JHI-Hv50k-2016-413564 on chromosome 6H which is part of ‘LD Block 6H - 488’ and associated with all four traits is highlighted by the dashed red line. The grey dashed lines correspond to two other LD-blocks associated with the traits RFW and Net.Area.

After removing the duplicated markers found for different traits or with different methods, 260 unique loci (markers or LD blocks) were found to be associated (p-value_FDR_< 0.1) with phenotypic traits under the control conditions, osmotic stress conditions and/or their ratios. Among the unique loci, 38 loci were associated with more than a single phenotypic trait (**Supplementary Table S11**). Three of these multi-trait loci were associated with four phenotypic traits. ‘JHI-Hv50k-2016-413564’ on chromosome 6H (within the 29 kb long ‘LD block 6H - 488’, which includes four highly linked SNP markers), displayed associations with RDW, RFW, Net.Area, and TotalBio under control (**Figure 3**). The marker ‘JHI-Hv50k-2016-187836’ was found to be associated with Net.Area-PEG, Net.Dep-PEG, Net-Len-PEG, and the Net.Area-stress/control-ratio under osmotic stress. Furthermore, ‘LD block 5H - 13’ has been shown to be linked to RFW-PEG, RDW-PEG, and four ratio traits. An additional set of four loci ‘JHI-Hv50k-2016-514383’, ‘SCRI_RS_223100’, ‘LD block 4H - 196’, and the large ‘LD block 7H - 752’ (spanning a region of 1.2 Mb and containing 38 markers on chromosome 7H) were each associated with three phenotypic traits, respectively (**Supplementary Table S10**). These seven loci associated with three to four traits are referred to as hotspot QTL.

For 11 of the 16 ratio traits, significant or at least potentially interesting associations could be detected (**Supplementary Table S11**). Among the loci identified to be associated with the ratio traits, 15 were multi-trait loci associated with more than a single trait. Four loci, ‘JHI-Hv50k-2016-187836’, ‘LD block 5H-13’, ‘SCRI_RS_223100’, and ‘LD block 4H-196’ were categorized as hotspot QTL being associated with at least three traits (**Supplementary Table S12**). The first locus ‘JHI-Hv50k-2016-187836’ displayed high associations with Net.Area under osmotic stress conditions, detected by FarmCPU and BLINK, as well as with the Net.Area-stress/control-ratio and two other traits: Net.Dep-PEG and Net.Len-PEG. The second locus ‘LD block 5H - 13’, showed association with root fresh weight and root dry weight under stress conditions and the corresponding RDW-stress/control-ratio. In addition, this locus was identified to be associated with three more ratio traits: Net.Area-stress/control-ratio, Net.Dep-stress/control-ratio and TotalBio-stress/control-ratio. The third locus ‘SCRI_RS_223100’, is highly associated with the trait Net.Dep under stress conditions and the two ratio traits for Net.Area-stress/control-ratio and Net.Len-stress/control-ratio, while the fourth locus ‘LD block 4H - 196’ has been found to be associated with Net.Area, Net.Dep-PEG and the corresponding Net.Dep-stress/control-ratio.

### Candidate genes

**Figure 4:**
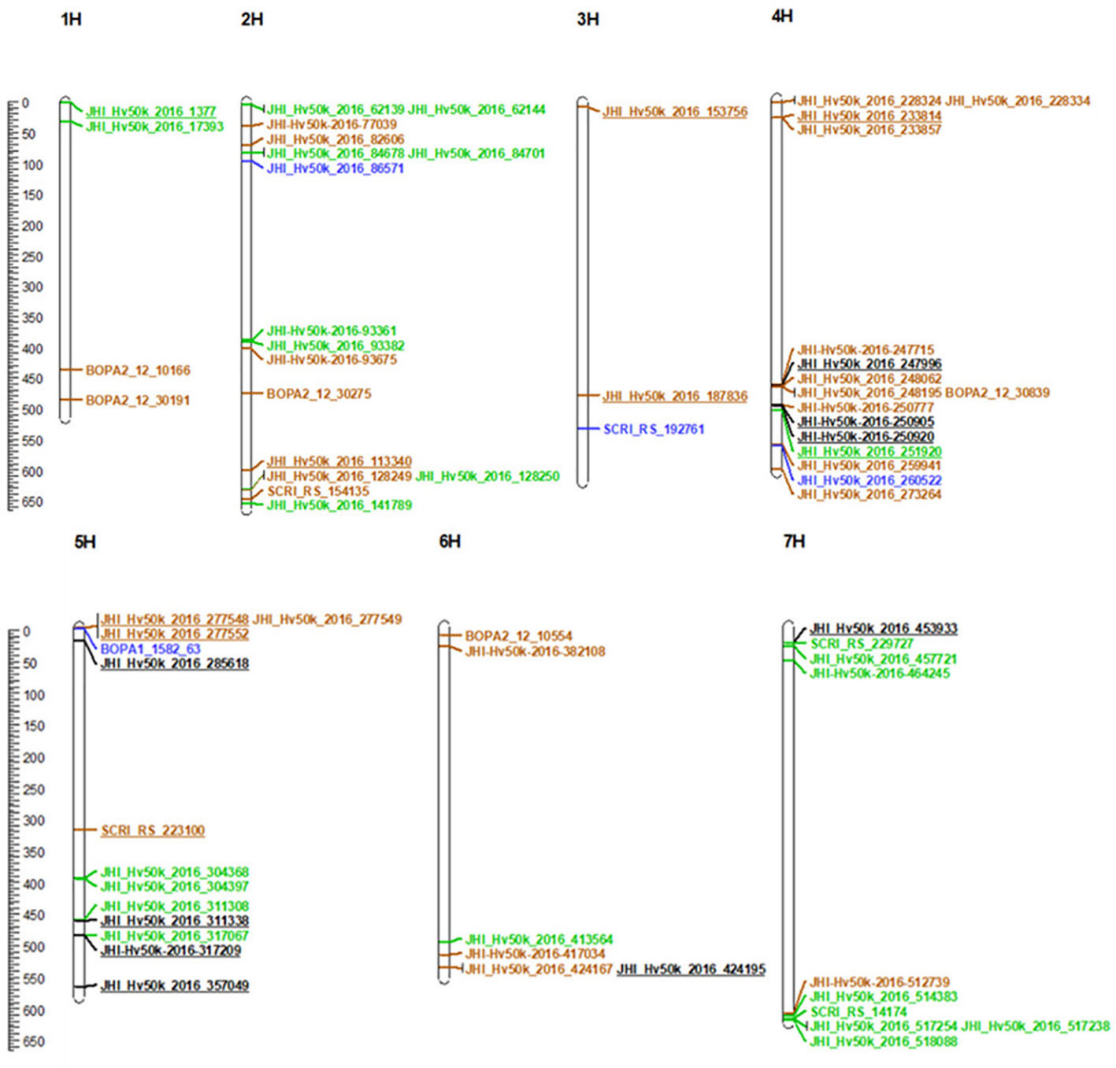
Location of QTL projected on the barley Morex V3 reference sequence assembly. All 68 markers from 52 common and multi-trait LD blocks were mapped on the barley Morex V3 reference sequence assembly. In green: markers associated with control, in brown: markers associated with PEG, in blue: markers associated both with control and PEG, in dark and/or underlined: markers associated with ratio. The map was drawn with MapChart 2.32 Windows (Voorrips, 2002). Chromosome length and marker positions are indicated by the scale on the left side in Mbp.

A total of 52 genomic regions were inspected in detail to identify candidate genes, whereby the search intervals around the associated markers/LD blocks were extended to the flanking genetic markers. Twenty seven of these regions were identified by at least two of the GWAS methods (**Table 2**) and 38 of them were associated with more than one phenotypic trait (**Supplementary Table S11**) under osmotic stress, the control conditions, and/or their ratio. Only genes annotated with a high confidence were considered as candidate genes with putative a role in barley root growth. In this context, no candidate genes could be nominated for 17 of these regions. In total, 273 candidate genes were identified for the 35 remaining regions. Five regions were large LD blocks (2H_354, 5H_449, 2H_351, 2H_247 and 7H_752) containing 23, 18, 17, 60 and 29 genes, respectively. A comprehensive list of all candidate genes is given in **Supplementary Table S12**. Of the 273 genes potentially linked to root growth under control and stress conditions, 163 could not be attributed a BIN functional category. It is noteworthy that the same genes had an undefined GO annotation (**Supplementary Table S12**). Whereas some categories were represented in both treatments, others were specific either for control or osmotic stress conditions (**Figure S6**). BIN categories specific to stress-associated traits included, but were not restricted to, ‘Cellular respiration/pyruvate oxidation’ (HORVU.MOREX.r3.2HG0121000), ‘Lipid metabolism’ (HORVU.MOREX.r3.1HG0079910), ‘Redox homeostasis/thiol-based redox regulation’ (HORVU.MOREX.r3.2HG0120840) or ‘Cell wall organisation’ (HORVU.MOREX.r3.4HG0403060). Those specific genes have been previously described to have a role in response to stress in different species (Sevilla et al., 2015; Wang et al., 2021). Interestingly, we observed that regions of interest, and consequently genes, putatively associated with growth parameters in control conditions were mostly localized on chromosomes 2, 5 and 7, whereas regions associated with growth parameters under osmotic stress were mostly localized on the chromosomes 1, 2, 3 and 4 (**Figure 4**).

## Discussion

### Phenotypic variation of RSA and shoot traits

In nature, drought can occur at any stage of the plant’s life cycle and drastically affect plant growth and development (Khodaeiaminjan and Bergougnoux, 2021). Drought tolerance is a complex response involving both shoot and root adaptation. Landraces have been evaluated for centuries in different regions of the world in traditional agrosystems where they adapted to specific agroclimatic conditions while representing significantly broader genetic diversity between and within populations than modern varieties. Therefore, landraces constitute an important genetic resource for new breeding programs. In this study using a population of 230 individual two-row spring barley landraces, we simulated drought shortly after seed germination. Plants were grown in germination pouches, as described in several studies (Acharya et al., 2017; Huang et al., 2020), especially for root development under drought (Richard et al., 2015; Couchoud et al., 2019). High degrees of variation for all shoot and root traits were observed in both osmotic-stress and control conditions. Among the 16 traits under control conditions, the highest heritability was observed for SDW, in line with previous studies (Abdel-Ghani et al., 2019; Jia et al., 2019). Comparison of phenotypic traits between hulled and naked landraces revealed great differences between root and shoot traits under stress and control conditions. In general, hulled genotypes showed higher agronomic trait values under both conditions. However, some traits such as SDW were higher in naked genotypes. An influence of caryopsis and row type on yield and phenotypic traits has been reported previously (Jui et al., 1997; Jia et al., 2019). Indeed, naked barley closely resembles hulled type barley in its agronomic characteristics. Modern naked barley cultivars provided higher yield compared to classical, hulled barley cultivars, indicating that agronomic traits such as lower yield could be attributed more to the origin of genotypes and lack of adaptation rather than to the naked trait itself (Dickin et al., 2012). However, it has been proposed that the higher risk of embryo damage in naked types is responsible for lower yield compared to hulled types (Choo et al., 2001).

### Identifying novel QTL and putative candidate genes associated with root and shoot traits

Drought signalling and plant response to drought are complex mechanisms, controlled by multiple loci. Plants develop different morphological and physiological strategies to cope with water deficit, and roots play a key role in drought adaptation. For instance, a deeper root system that enables access to moisture in deeper soil layers (Pérez-Ramos et al., 2013) and a higher root/shoot ratio are important adaptation traits to water deficit conditions (Jiang et al., 2012). To date, only few studies identified QTL associated with RSA in barley under water deficit. Robinson et al. (2016) assessed the seminal root angle and number under control conditions in soil-grown barley. Jia et al. (2019) reported 55 QTL associated with RSA variation in barley under well-controlled greenhouse conditions. Abdel-Ghani et al. (2019) identified 34 root-specific loci, in a spring barley collection under osmotic stress and control condition using GWAS. In the present study, conducted solely on barley landraces, we identified a total of 276 MTAs (p-value (FDR)≤0.05) representing 210 unique genetic markers/LD blocks associated with RSA, biomass, and shoot traits in both osmotic-stress and control conditions. Using the latest barley reference genome version (Morex v3; Mascher et al., 2021), we observed that marker positions changed in comparison to previous studies. Nevertheless, in total, the present study shares 13 loci with genomic regions detected by Abdel-Ghani et al (2019); **Supplementary Table S13**). Four were classified as hotspot QTL, six were classified as root-specific QTL, one was classified as stress-specific and two as non-specific QTL. Our hotspot QTL in LD block 2H-259 coincided with hotspot QTL-2H-6 of Abdel-Ghani et al. (2019): the authors suggested *Thioredoxin-m3* (*TRX-m3*) as a potential candidate gene in this region. The role of *TRX-m3* in root meristem and root hairs development has been shown in Arabidopsis previously (Benitez-Alfonso and Jackson, 2009). Further, our hotspot QTL in LD block 4H-316 was in the same genomic region as the hotspot QTL-4H-4 of Abdel-Ghani et al. (2019), and *HvPRR73* was suggested to be a candidate gene for this locus. *HvPRR37* induces early flowering under long photoperiods in barley (Turner et al., 2005). Previously, upregulation of *HvPRR37* under osmotic stress was reported in barley seedlings (Habte et al., 2014). Moreover, hotspot QTL-5H-1 of Abdel-Ghani et al. (2019) coincided with our hotspot QTL in LD block 5H-137. Also, we mapped LD block 7H -752 to the large hotspot genomic region QTL-7H-10 of Abdel-Ghani et al. (2019) and in its later proximity *HvDIM* is a suggested candidate gene. *HvDIM* is involved in the brassinosteroid biosynthetic in barley (Dockter et al., 2014). Brassinosteroids play role in cell division, cell elongation, and photosynthesis (Gudesblat and Russinova, 2011). Further, the present study detected QTL for root traits in the vicinity of root-specific QTL in the study of Abdel-Ghani et al. (2019): QTL-1H-4 (BOPA2_12_10166 associated with Net.Con.A-PEG, Net.Len-PEG in our study), QTL-2H-10 (JHI-Hv50k-2016-113340 associated with Net.Area-PEG, Net.Len-PEG_CONTROL-ratio in our study), QTL-3H-3 (JHI-Hv50k-2016-187836 associated with Net.Area-PEG, Net.Len-PEG, Net.Dep-PEG, Net.Area-PEG_CONTROL-ratio in our study), QTL-4H-6 (JHI-Hv50k-2016-260522 associated with Net.Solid-PEG, RFW in our study), QTL-5H-2 (SCRI_RS_223100 associated with Net.Dep-PEG, Net.Area-PEG_CONTROL-ratio, Net.Len-PEG_CONTROL-ratio in our study), and QTL-5H-3 (JHI-Hv50k-2016-311338 associated with RFW-PEG_CONTROL-ratio in our study). The marker BOPA2_12_10166 detected for root traits in our study in the vicinity of root and stress specific QTL-1H-4 found by Abdel-Ghani et al. (2019) was also in our study exclusively associated with root traits from the stress treatment and therefore the QTL region can be confirmed as a stress and root specific in diverse spring barley with high importance for harboring a candidate gene influencing root growth under osmotic stress.

In opposite to the previous studies, we did not detect any root or shoot QTL in the region of the main flowering time gene in spring barley, HvPpd-H1 (Tombuloglu, 2019), although our barley panel differs for photoperiod sensitivity. This attributes to the fact that our experiments were conducted in day neutral conditions.

### QTL and genes associated with growth in control condition

Net.Area, representing the surface of the root network, is an important morphological root trait in crop productivity under drought conditions (Vadez et al., 2013). It was found to be associated with 5 LD blocks (7H-243, 6H-488, 2H-259, 2H-351, 7H-752), mostly located on chromosome 2 or 7, either alone or together with traits such as root fresh/dry weight, total biomass, or root number. The LD block 7H-752 is characterized by 29 genes, including five genes encoding F-box transcription factors involved in a plethora of physiological and developmental processes, including promotion of lateral root (Lechner et al., 2006; Iantcheva et al., 2015), a gene encoding the gibberellin-regulated protein 2/Gibberellic Acid-Stimulated in Arabidopsis (GASA), and a gene encoding a phytoene synthase. GASA proteins trigger phytohormone-responses, such as cell wall and lateral root development while contributing to root primordium formation (Zimmermann et al., 2009; Li et al., 2011; Ahmad et al., 2019). Phytoene synthases catalyse the rate-limiting step of carotenoid, abscisic acid precursor, and strigolactone (SL) biosynthesis. SL regulate root development and possibly also root meristem cell number (Kapulnik and Koltai, 2014) (Sun et al., 2016). LD block 2H-259 contains three genes: two encoding glycosyltransferases and a lipase/lipooxygenase. Glycosyltransferases are involved in the biosynthesis of polysaccharides and glycoproteins in the plant cell wall (Hansen et al., 2012). Overexpressing the saffron UDP-glucosyltransferase increased anchoring root development in Arabidopsis (Ahrazem et al., 2015). Lipase/lipooxygenase, PLAT/L2H, seems to be involved in plant growth and abiotic stress tolerance, as overexpression of PLAT1 in Arabidopsis, increased abiotic stress tolerance, including cold, drought and salt stresses, while under control condition PLAT1 promoted growth (Hyun et al., 2014). LD block 2H-351 contains 17 high confidence genes. Two prime candidates are the histidine kinase 5, a receptor involved in the signalling pathway of cytokinins (CKs) and a laccase. In Arabidopsis, it has been demonstrated that HK5 is involved in root elongation, (Iwama et al., 2006), working antagonistically to ABA and ethylene. Laccases are enzymes involved in oxidative polymerization of lignin that accumulates particularly in cells where secondary thickening of cell wall occurs (Zhao et al., 2013). The importance of laccase in root hairs and xylem development has been demonstrated in *Salvia miltiorrhiza* (Zhou et al., 2021). Interestingly two tandem arrayed laccases were also located within the LD block 6H-488 that contains four high confidence genes. Two recent studies identified *LAC2* as a gene improving drought tolerance in *Populus euphratica* by enhancing water transport capacity (Niu et al., 2021), and as a negative regulator of lignin deposition in the root xylem tissue of Arabidopsis thaliana (Sharma et al., 2020). It is interesting to note that in this LD block, the two laccases are associated with the exocyst complex component SEC6. Exocysts are protein complexes tethering secretory vesicles to target membranes; they function in plant cell secretory pathways, notably during cell wall biogenesis (Žárský et al., 2013). Genes of the laccase family therefore represent promising targets for breeding programs.

The trait Net.Len, representing the maximum deepness of the root system, corresponds to the foraging ability of the seedling and its ability to develop roots deep in the substrate. The genetic marker JHI-Hv50k-2016-93361 is associated with Net.Len and the corresponding search interval is characterized by several genes whose function in root growth has already been reported. The NAC (NO APICAL MERISTEM, ACTIVATION FACTOR, and CUP-SHAPED COTYLEDON) transcription factor (TF) family represents one of the largest plant TF families. NACs control lateral root formation *via* auxin signaling (Xie et al., 2000). Interestingly, an increase in root number and root diameter was observed in plants overexpressing OsNAC6, resulting in improved drought tolerance (Lee et al., 2017). NACs enhance drought tolerance in rice by transcriptional control of a subset of downstream genes (Hong et al., 2016).

The LD block 5H-449 associated with RFW encompasses 18 genes. The biochemical functions of these genes are highly variable. A role in biomass accumulation has been reported for MIZU-KUSSEI1 as a positive regulator of hydrotropic response in plants (Miyazawa et al., 2012). In rice, a differential expression of MIZ1 was observed between drought-tolerant and sensitive genotypes (Kaur et al., 2020). Furthermore, MIZ1 contributed to a higher survival rate in drought conditions in Arabidopsis (Iwata et al., 2013).

The LD block 4H-356, associated with RDW, covers two genes. One of those genes, encoding the phytochrome interacting factor 3 (PIF3). PIF3 is a component of the phytochrome signalling pathway, mediating response to red light. In *Picea abies*, red light has been shown to controls adventitious root regeneration by modulating hormone homeostasis (Alallaq et al., 2020).

The genetic marker JHI-Hv50k-2016-317067 was detected to be associated with the trait Net.width. Seven genes were associated with this QTL. However, except of TPX2 and ubiquitin hydrolase, none of them were described in plants.

### QTL and genes associated with growth under osmotic stress conditions

We observed little overlap between control- and osmotic stress-related QTL: four genetic markers were related to RSR (SCRI_RS_121978, SCRI_RS_211274, SCRI_RS_152795 and BOPA1_1582-63) and one was related to SDW (SCRI_RS_192761). QTL associated with growth parameters under osmotic stress were mostly localized on chromosomes 1, 2, 3 and 4. The correlation between high leaf RWC and drought stress resistance in crops has been known for a long time (Schonfeld et al., 1988). The genetic marker JHI-Hv50k-2016-153756 was associated with RWC under stress condition. The corresponding search interval contained four genes encode trypsin inhibitors. Under water deficit, trypsin inhibitors regulate protein metabolism and contribute to maintain RWC (Dramé et al., 2013; Vaseva et al., 2016; Malefo et al., 2020). JHI-Hv50k-2016-273264 on chromosome 4H was identified by all three GWAS methods for SDW. Only one gene, virulence factor-related M protein that might play important roles in the mechanisms of virulence over the pathogen infection cycle was linked to this marker (Figaj et al., 2019).

Five genetic markers/LD blocks were identified for Net.Area-PEG alone or associated with different PEG-traits. LD block 4H-465 is contains a gene encoding a protein with crucial roles in fatty acid metabolism. Fatty acyl-CoA reductases have been reported to be involved in primary alcohol biosynthesis as the major components of cuticular wax on wheat seedling leaves (Wang et al., 2015). In wheat seedlings the expression of genes encoding fatty acyl-coenzyme A reductases, increased in response to dehydration stress (Chai et al., 2018). The genetic marker JHI-Hv50k-2016-113340 is associated with two high confidence genes. The gene encoding a DJ-1 protein regulates oxidative stress by interacting with the antioxidant enzymes glutathione peroxidase and superoxide dismutase in Arabidopsis (Xu et al., 2010). Mohanan et al. (2020) showed that overexpressing a gene containing a DJ-1 domain improved drought tolerance in sugarcane, however no effects on root anatomy were observed. Armadillo/beta-catenin-like repeat family proteins control lateral root branching (Gardiner et al., 2011) and are involved in stress signalling under abiotic stress (Sharma et al., 2014).

LD block 2H-370 is associated with Max.NR-PEG and includes six genes. The TF abscisic acid-stress-ripening (ASR) is a key regulator of plant drought responses (Virlouvet et al., 2011) (González and Iusem, 2014) (Sachdeva et al., 2020). The accumulation of ASR transcripts varied in rice plants under water stress, depending on the types of root tissue, suggesting a main role in root architecture changes (Yang et al., 2004). COP9 signalosome is a multiprotein complex formed by eight subunits and controls plant development and hormone signalling, especially auxin (Singh and Chamovitz, 2019). In Arabidopsis, the subunit 4 regulates adventitious root formation (Pacurar et al., 2017). Ubiquitin carboxyl-terminal hydrolases are involved in ubiquitin regeneration and protein stability and were identified in a miRNA study in barley roots under salt stress (Kuang et al., 2019), and are upregulated in drought tolerant maize (Dong et al., 2020).

LD block 2H-247, containing 60 genes, is associated with RFW-PEG and TotalBio-PEG. The TFs containing the AT-hook motif (AHL) regulate growth under stress (Wong et al., 2019) (Wang et al., 2021). In rice, overexpression of OsAHL1 improved root volume under water stress and this gene also regulated chlorophyll content of the leaves (Zhou et al., 2016). We detected a putative glycosyltransferase, the β -1,3-n-acetylglucosaminyltransferase radical fringe protein. β-glucosidase 2 (GBA2) is one of the main enzymes involved in degradation of cell wall. GBA2 was upregulated under water stress in susceptible maize plants (Waititu et al., 2021). DnaJ chaperones, involved in assisting protein folding, were also upregulated under PEG-induced water stress in tobacco (Xia et al., 2014) and tomato (Wang et al., 2019). Cullin-associated NEDD8-dissociated protein 1 (CAND1), are involved in AUX signaling and have been reported to regulate rice crown root emergence (Wang et al., 2011), tomato root architecture, and vegetative growth in general (Cheng et al., 2020). Alpha-L-arabinofuranosidase 1 is involved in cell wall metabolism and root elongation processes (Kozlova et al., 2015). It accumulates in common bean (Zadražnik et al., 2017) and chilli leaves (Jaswanthi et al., 2019) under drought stress. Kinesin-like proteins interact with tubulins and have been described to be accumulated under drought stress in mycorrhizal roots of wheat (Bernardo et al., 2017). Flavin-containing monooxygenase (FMO) control the metabolism of glucosinolates and auxin biosynthesis (Shi et al., 2014). It has been reported that in wheat FMOs are involved in the root acclimation to drought (Grzesiak et al., 2019). Another protein regulating redox homeostasis present in this block is Glutaredoxin (GRX), a glutathione-dependent reductase. Overexpression of the tomato homologue *SlGRX* in Arabidopsis improved the tolerance to water stress (Guo et al., 2010). Moreover, longer roots were observed in transgenic Arabidopsis overexpressing *OsGRX* under water stress conditions (Kumar et al., 2020b). MYB TFs control plant development, cell fate, production of metabolites and plant stress responses (Dubos et al., 2010). In wheat expression of *TaMYB31* was upregulated under PEG or ABA treatment and altered the expression of wax biosynthetic genes and genes responsive to drought stress (Zhao et al., 2018). In Arabidopsis, AtMYB068/AtMYB084 functions in root elongation. AtMYB017/AtMYB023 and AtMYB066 have functions in root hair patterning and development (Ambawat et al., 2013). P-loop containing nucleoside triphosphate hydrolases are involved in many functions, including cell division and signal transduction (Leipe et al., 2004). This gene was related to water potential and yield under water deficit in Arabidopsis (Verslues et al., 2014) and potato (Tagliotti et al., 2021).

The interval around the genetic marker BOPA2-12-30191, associated with SDW-PEG and SFW-PEG contains 13 genes, with an overrepresentation of pectin lyase-like superfamily proteins and MYB transcription factors. It contains also a gene encoding a farnesyl disphophate synthase, involved in the synthesis of secondary metabolites, such as sterols (Szkopińska and Płochocka, 2005). Sterols are essential components of the cell membrane. Their accumulation under drought stress in different species (Du et al., 2020) could be part of the mechanism to protect membrane integrity.

LD block 5H-13 was associated with both, RFW-PEG and RDW-PEG. It contains four genes, including FtsH, which accumulates in drought-tolerant chickpea and maize (Vessal et al., 2020; Zhang et al., 2020) and a cation/H (+) antiporter classified by Mercator as belonging to the CPA-2 family. A genome-wide study in radish demonstrated that CPAs are accumulating in different tissues and in response to various abiotic stresses (Wang et al., 2020).

The genetic marker BOPA2-12-10166 was detected for Net.Con.A-PEG and Net.Len-PEG and its corresponding search interval contains five genes, four of them encoding wound-induced protease inhibitors (PI). Several studies supported that PI are involved in response to abiotic stresses, including drought (Shan et al., 2008; Srinivasan et al., 2009; Mishra et al., 2012).

## Conclusions

In the present study, using a spring barley landrace collection, we identified QTL involved in barley root system architecture both in control and osmotic stress conditions. It is noteworthy that different regions of the genome are involved in either growth in normal condition or growth under stress. Some QTL overlapped regions that were already described to be associated with root development, whereas some others are unique as they were not described yet. This might be attributed to higher genetic diversity represented in the landraces compared to cultivars used in different studies. This highlights the high potential of such older genetic material for future breeding programs and the development of new varieties with higher foraging activity towards more efficient water and nutrient uptake, mobilization, and usage. Deep or highly branched root system ensure sufficient plant nutrition and maintenance of yield under drought stress conditions. Water deficit inhibits primary root growth, which is accompanied by chemical modification of cell wall, including lignin’s deposition in different species (Yang et al., 2004; Pospíšilová et al., 2016; Sharma et al., 2020). Due to its hydrophobic nature, it is assumed that highly lignified cell walls are less permeable to water, preventing thus water leakage and supporting water and nutrient’s transport under stress conditions. Some important root traits such as RFW, Net.Area and MaxNR were associated with QTL encompassing genes involved in cell wall organization(production, accumulation and modification of cell wall). However, the function of these promising candidate genes during root development in response to stress will have to be validated by different approaches, including gene expression analyses, histochemical studies of root anatomy, and overexpression or targeted gene knockout using CRISPR/Cas9.

## Data availability statement

The original contributions presented in the study are included in the article/**Supplementary Material**. Further inquiries can be directed to the corresponding authors. The genomic data for this study have been deposited in the European Variation Archive (EVA) at EMBL-EBI under accession number PRJEB59438 (https://www.ebi.ac.uk/eva/?eva-study=PRJEB59438).

## Author contributions

MK, KN, CFM and VB designed the experiments. MK, VB, CFM, NK, AT and TDN performed the experiments. MRT, VB and PG analysed the images; VaB and ID developed the macro for image analysis. MK, DK, JC, and KN analysed the data and performed GWAS. KN and AG provided seed material and genotypic data. VB managed the project and advised on interpretation. MK wrote the manuscript draft with the contribution of CFM, NK, AT and TDN. KN, DK, VB and PG edited the manuscript. All authors agree with the manuscript.

## References

[B1] Abdel-Ghani A. H. Sharma R. Wabila C. Dhanagond S. Owais S. J. Duwayri M. A.. (2019). Genome-wide association mapping in a diverse spring barley collection reveals the presence of QTL hotspots and candidate genes for root and shoot architecture traits at seedling stage. BMC Plant Biol. 19, 216. doi: 10.1186/s12870-019-1828-5 31122195 PMC6533710

[B2] Acharya B. R. Roy Choudhury S. Estelle A. B. Vijayakumar A. Zhu C. Hovis L.. (2017). Optimization of phenotyping assays for the model monocot setaria viridis. Front. Plant Sci. 8. doi: 10.3389/fpls.2017.02172 PMC5743732 29312412

[B3] Ahmad M. Z. Sana A. Jamil A. Nasir J. A. Ahmed S. Hameed M. U.. (2019). A genome-wide approach to the comprehensive analysis of GASA gene family in glycine max. Plant Mol. Biol. 100, 607–620. doi: 10.1007/s11103-019-00883-1 31123969

[B4] Ahrazem O. Rubio-Moraga A. Trapero-Mozos A. Climent M. F. L. Gómez-Cadenas A. Gómez-Gómez L. (2015). Ectopic expression of a stress-inducible glycosyltransferase from saffron enhances salt and oxidative stress tolerance in arabidopsis while alters anchor root formation. Plant Sci. 234, 60–73. doi: 10.1016/j.plantsci.2015.02.004 25804810

[B5] Alallaq S. Ranjan A. Brunoni F. Novák O. Lakehal A. Bellini C. (2020). Red light controls adventitious root regeneration by modulating hormone homeostasis in picea abies seedlings. Front. Plant Sci. 11. doi: 10.3389/fpls.2020.586140 PMC7509059 33014006

[B6] Ambawat S. Sharma P. Yadav N. R. Yadav R. C. (2013). MYB transcription factor genes as regulators for plant responses: an overview. Physiol. Mol. Biol. Plants 19, 307–321. doi: 10.1007/s12298-013-0179-1 24431500 PMC3715649

[B7] Anscombe F. J. Tukey J. W. (1963). The Examination and Analysis of Residuals. Technometrics 5 (2), 141–160. doi: 10.1080/00401706.1963.10490071

[B8] Barrs H. D. Weatherley P. E. (1962). A re-examination of the relative turgidity technique for estimating water deficits in leaves by h. d. barrs* and p. e. weatherleyt. Aust. J. Biol. Sci 15 (3), 413–428. doi: 10.1071/BI9620413

[B9] Bayer M. M. Rapazote-flores P. Ganal M. Hedley P. E. Macaulay M. Plieske J.. (2017). Development and evaluation of a barley 50k iSelect SNP array. Front. Plant Sci. 8, 1–10. doi: 10.3389/fpls.2017.01792 29089957 PMC5651081

[B10] Benitez-Alfonso Y. Jackson D. (2009). Redox homeostasis regulates plasmodesmal communication in arabidopsis meristems. Plant Signaling Behav. 4, 655–659. doi: 10.4161/psb.4.7.8992 PMC2710567 19820302

[B11] Benjamini Y. Hochberg Y. (1995). Controlling the false discovery rate: A practical and powerful approach to multiple testing. J. R. Stat. Society. Ser. B (Methodological) 57, 289–300. doi: 10.1111/j.2517-6161.1995.tb02031.x

[B12] Bernardo L. Morcia C. Carletti P. Ghizzoni R. Badeck F. W. Rizza F.. (2017). Proteomic insight into the mitigation of wheat root drought stress by arbuscular mycorrhizae. J. Proteomics 169, 21–32. doi: 10.1016/j.jprot.2017.03.024 28366879

[B13] Browning S. R. Browning B. L. (2007). Rapid and accurate haplotype phasing and missing-data inference for whole-genome association studies by use of localized haplotype clustering. Am. J. Hum. Genet. 81, 1084–1097. doi: 10.1086/521987 17924348 PMC2265661

[B14] Browning B. L. Zhou Y. Browning S. R. (2018). A one-penny imputed genome from next-generation reference panels. Am. J. Hum. Genet. 103, 338–348. doi: 10.1016/j.ajhg.2018.07.015 30100085 PMC6128308

[B15] Chai G. Li C. Xu F. Li Y. Shi X. Wang Y.. (2018). Three endoplasmic reticulum-associated fatty acyl-coenzyme a reductases were involved in the production of primary alcohols in hexaploid wheat (Triticum aestivum l.). BMC Plant Biol. 18, 41. doi: 10.1186/s12870-018-1256-y 29506473 PMC5836450

[B16] Cheng W. Yin S. Tu Y. Mei H. Wang Y. Yang Y. (2020). SlCAND1, encoding cullin-associated Nedd8-dissociated protein 1, regulates plant height, flowering time, seed germination, and root architecture in tomato. Plant Mol. Biol. 102, 537–551. doi: 10.1007/s11103-020-00963-7 31916084

[B17] Choo T. Ho K. M. Martin R. A. (2001). Genetic analysis of a hulless × covered cross of barley using doubled-haploid lines. Crop Sci. 41, 1021–1026. doi: 10.2135/cropsci2001.4141021x

[B18] Colmsee C. Beier S. Himmelbach A. Schmutzer T. Stein N. Scholz U.. (2015). BARLEX – the barley draft genome explorer. Mol. Plant 8, 964–966. doi: 10.1016/j.molp.2015.03.009 25804976

[B19] Couchoud M. Der C. Girodet S. Vernoud V. Prudent M. Leborgne-Castel N. (2019). Drought stress stimulates endocytosis and modifies membrane lipid order of rhizodermal cells of medicago truncatula in a genotype-dependent manner. BMC Plant Biol. 19, 221. doi: 10.1186/s12870-019-1814-y 31138155 PMC6537417

[B20] Del Bianco M. Kepinski S. (2018). Building a future with root architecture. J. Exp. Bot. 69, 5319–5323. doi: 10.1093/jxb/ery390 30445468 PMC6255693

[B21] Dickin E. Steele K. Edwards-Jones G. Wright D. (2012). Agronomic diversity of naked barley (Hordeum vulgare l.): a potential resource for breeding new food barley for Europe. Euphytica 184, 85–99. doi: 10.1007/s10681-011-0567-y

[B22] Dockter C. Gruszka D. Braumann I. Druka A. Druka I. Franckowiak J.. (2014). Induced variations in brassinosteroid genes define barley height and sturdiness, and expand the green revolution genetic toolkit. Plant Physiol. 166, 1912–1927. doi: 10.1104/pp.114.250738 25332507 PMC4256852

[B23] Dong A. Yang Y. Liu S. Zenda T. Liu X. Wang Y.. (2020). Comparative proteomics analysis of two maize hybrids revealed drought-stress tolerance mechanisms. Biotechnol. Biotechnol. Equip. 34, 763–780. doi: 10.1080/13102818.2020.1805015

[B24] Dramé K. N. Passaquet C. Repellin A. Zuily-Fodil Y. (2013). Cloning, characterization and differential expression of a bowman–birk inhibitor during progressive water deficit and subsequent recovery in peanut (Arachis hypogaea) leaves. J. Plant Physiol. 170, 225–229. doi: 10.1016/j.jplph.2012.09.005 23084322

[B25] Du Y. Zhao Q. Chen L. Yao X. Zhang W. Zhang B.. (2020). Effect of drought stress on sugar metabolism in leaves and roots of soybean seedlings. Plant Physiol. Biochem. 146, 1–12. doi: 10.1016/j.plaphy.2019.11.003 31710920

[B26] Dubos C. Stracke R. Grotewold E. Weisshaar B. Martin C. Lepiniec L. (2010). MYB transcription factors in arabidopsis. Trends Plant Sci. 15, 573–581. doi: 10.1016/j.tplants.2010.06.005 20674465

[B27] Ehdaie B. Layne A. P. Waines J. G. (2012). Root system plasticity to drought influences grain yield in bread wheat. Euphytica 186, 219–232. doi: 10.1007/s10681-011-0585-9

[B28] Evanno G. Regnaut S. Goudet J. (2005). Detecting the number of clusters of individuals using the software structure: a simulation study. Mol. Ecol. 14, 2611–2620. doi: 10.1111/j.1365-294X.2005.02553.x 15969739

[B29] Fang C. Ma Y. Wu S. Liu Z. Wang Z. Yang R.. (2017). Genome-wide association studies dissect the genetic networks underlying agronomical traits in soybean. Genome Biol. 18, 161. doi: 10.1186/s13059-017-1289-9 PMC5571659 28838319

[B30] Figaj D. Ambroziak P. Przepiora T. Skorko-Glonek J. (2019). The role of proteases in the virulence of plant pathogenic bacteria. IJMS 20, 672. doi: 10.3390/ijms20030672 30720762 PMC6386880

[B31] Galkovskyi T. Mileyko Y. Bucksch A. Moore B. Symonova O. Price C. A.. (2012). GiA roots: Software for the high throughput analysis of plant root system architecture. BMC Plant Biol. 12, 116. doi: 10.1186/1471-2229-12-116 22834569 PMC3444351

[B32] Gardiner J. Overall R. Marc J. (2011). Plant microtubule cytoskeleton complexity: microtubule arrays as fractals. J. Exp. Bot. 63, 635–642. doi: 10.1093/jxb/err312 22016422

[B33] Geng D. Chen P. Shen X. Zhang Y. Li X. Jiang L.. (2018). MDMYB88 and MDMYB124 enhance drought tolerance by modulating root vessels and cell walls in apple. Plant Physiol. 178, 1296–1309. doi: 10.1104/pp.18.00502 30190418 PMC6236628

[B34] Gilmour A. R. Thompson R. Cullis B. R. (1995). Average information REML: An efficient algorithm for variance parameter estimation in linear mixed models. Biometrics 51 (4), 1440–1450. doi: 10.2307/2533274

[B35] González R. M. Iusem N. D. (2014). Twenty years of research on asr (ABA-stress-ripening) genes and proteins. Planta 239, 941–949. doi: 10.1007/s00425-014-2039-9 24531839

[B36] Grzesiak M. T. Hordyńska N. Maksymowicz A. Grzesiak S. Szechyńska-Hebda M. (2019). Variation among spring wheat (Triticum aestivum l.) genotypes in response to the drought stress. II–root system structure. Plants 8 (12), 584. doi: 10.3390/plants8120584 31817986 PMC6963452

[B37] Gudesblat G. E. Russinova E. (2011). Plants grow on brassinosteroids. Curr. Opin. Plant Biol. 14, 530–537. doi: 10.1016/j.pbi.2011.05.004 21802346

[B38] Guo Y. Huang C. Xie Y. Song F. Zhou X. (2010). A tomato glutaredoxin gene SlGRX1 regulates plant responses to oxidative, drought and salt stresses. Planta 232, 1499–1509. doi: 10.1007/s00425-010-1271-1 20862491

[B39] Habte E. Müller L. M. Shtaya M. Davis S. J. Von Korff M. (2014). Osmotic stress at the barley root affects expression of circadian clock genes in the shoot: Osmotic stress changes the barley circadian clock. Plant Cell Environ. 37, 1321–1337. doi: 10.1111/pce.12242 24895755

[B40] Hansen S. F. Harholt J. Oikawa A. (2012). And scheller, h Plant glycosyltransferases beyond CAZy: A perspective on DUF families V. Front. Plant Sci. 3. doi: 10.3389/fpls.2012.00059 PMC3355507 22629278

[B41] Hill W. G. Weir B. S. (1988). Variances and covariances of squared linkage disequilibria in finite populations. Theor. Popul Biol. 33, 54–78. doi: 10.1016/0040-5809(88)90004-4 3376052

[B42] Hong Y. Zhang H. Huang L. Li D. Song F. (2016). Overexpression of a stress-responsive NAC transcription factor gene ONAC022 improves drought and salt tolerance in rice. Front. Plant Sci. 7. doi: 10.3389/fpls.2016.00004 PMC4722120 26834774

[B43] Huang C.-T. Klos K. E. Huang Y.-F. (2020). Genome-wide association study reveals the genetic architecture of seed vigor in oats. G3 Genes|Genomes|Genetics 10, 4489–4503. doi: 10.1534/g3.120.401602 33028627 PMC7718755

[B44] Huang M. Liu X. Zhou Y. Summers R. M. Zhang Z. (2019). BLINK: a package for the next level of genome-wide association studies with both individuals and markers in the millions. GigaScience 8 (2). doi: 10.1093/gigascience/giy154 PMC6365300 30535326

[B45] Hyun T. K. van der Graaff E. Albacete A. Eom S. H. Großkinsky D. K. Böhm H.. (2014). The arabidopsis PLAT domain Protein1 is critically involved in abiotic stress tolerance. PloS One 9, e112946. doi: 10.1371/journal.pone.0112946 25396746 PMC4232524

[B46] Iantcheva A. Boycheva I. Vassileva V. Revalska M. Zechirov G. (2015). Cyclin-like f-box protein plays a role in growth and development of the three model species medicago truncatula, lotus japonicus, and arabidopsis thaliana. RRB 6, 117–130. doi: 10.2147/RRB.S84753 27180194

[B47] Iwama A. Yamashino T. Tanaka Y. Sakakibara H. Kakimoto T. Sato S.. (2006). AHK5 histidine kinase regulates root elongation through an ETR1-dependent abscisic acid and ethylene signaling pathway in arabidopsis thaliana. Plant Cell Physiol. 48, 375–380. doi: 10.1093/pcp/pcl065 17202180

[B48] Iwata S. Miyazawa Y. Fujii N. Takahashi H. (2013). MIZ1-regulated hydrotropism functions in the growth and survival of arabidopsis thaliana under natural conditions. Ann. Bot. 112, 103–114. doi: 10.1093/aob/mct098 23658369 PMC3690989

[B49] Jamieson P. D. Martin R. J. Francis G. S. (1995). Drought influences on grain yield of barley, wheat, and maize. New Z. J. Crop Hortic. Sci. 23, 55–66. doi: 10.1080/01140671.1995.9513868

[B50] Jaswanthi N. Krishna M. S. R. Sahitya U. L. (2019). Apoplast proteomic analysis reveals drought stress-responsive protein datasets in chilli (*Capsicum annuum* L.). Data Brief 25, 104041. doi: 10.1016/j.dib.2019.104041 31194032 PMC6546948

[B51] Jia Z. Liu Y. Gruber B. D. Neumann K. Kilian B. Graner A.. (2019). Genetic dissection of root system architectural traits in spring barley. Front. Plant Sci. 10. doi: 10.3389/fpls.2019.00400 PMC6454135 31001309

[B52] Jiang T. Fountain J. Davis G. Kemerait R. Scully B. Lee R. D.. (2012). Root morphology and gene expression analysis in response to drought stress in maize (Zea mays). Plant Mol. Biol. Rep. 30, 360–369. doi: 10.1007/s11105-011-0347-9

[B53] Jui P. Y. Choo T. M. Ho K. M. Konishi T. Martin R. A. (1997). Genetic analysis of a two-row × six-row cross of barley using doubled-haploid lines. Theor. Appl. Genet. 94, 549–556. doi: 10.1007/s001220050450

[B54] Kapulnik Y. Koltai H. (2014). Strigolactone involvement in root development, response to abiotic stress, and interactions with the biotic soil environment. Plant Physiol. 166, 560–569. doi: 10.1104/pp.114.244939 25037210 PMC4213088

[B55] Kaur V. Yadav S. K. Wankhede D. P. Pulivendula P. Kumar A. Chinnusamy V. (2020). Cloning and characterization of a gene encoding MIZ1, a domain of unknown function protein and its role in salt and drought stress in rice. Protoplasma 257, 475–487. doi: 10.1007/s00709-019-01452-5 31786672

[B56] Khodaeiaminjan M. Bergougnoux V. (2021). Barley grain development during drought stress: Current status and perspectives. Cereal Grains (IntechOpen), vol. 1. doi: 10.5772/intechopen.97183

[B57] Klie S. Nikoloski Z. (2012). The choice between MapMan and gene ontology for automated gene function prediction in plant science. Front. Gene. 3. doi: 10.3389/fgene.2012.00115 PMC3384976 22754563

[B58] Kozlova L. V. Gorshkov O. V. Mokshina N. E. Gorshkova T. A. (2015). And Differential expression of α-l-arabinofuranosidases during maize (Zea mays l.) root elongation. Planta 241, 1159–1172. doi: 10.1007/s00425-015-2244-1 25608890

[B59] Kristensen P. S. Jahoor A. Andersen J. R. Cericola F. Orabi J. Janss L. L.. (2018). Genome-wide association studies and comparison of models and cross-validation strategies for genomic prediction of quality traits in advanced winter wheat breeding lines. Front. Plant Sci. 9. doi: 10.3389/fpls.2018.00069 PMC5801407 29456546

[B60] Ksouri N. Jiménez S. Wells C. E. Contreras-Moreira B. Gogorcena Y. (2016). Transcriptional responses in root and leaf of prunus persica under drought stress using RNA sequencing. Front. Plant Sci. 7. doi: 10.3389/fpls.2016.01715 PMC5120087 27933070

[B61] Kuang L. Shen Q. Wu L. Yu J. Fu L. Wu D.. (2019). Identification of microRNAs responding to salt stress in barley by high-throughput sequencing and degradome analysis. Environ. Exp. Bot. 160, 59–70. doi: 10.1016/j.envexpbot.2019.01.006

[B62] Kumar A. Dubey A. K. Kumar V. Ansari M. A. Narayan S. Meenakshi. (2020a). Overexpression of rice glutaredoxin genes LOC_Os02g40500 and LOC_Os01g27140 regulate plant responses to drought stress. Ecotoxicology Environ. Saf. 200, 110721. doi: 10.1016/j.ecoenv.2020.110721 32464438

[B63] Kumar A. Verma R. P. S. Singh A. Kumar Sharma H. Devi G. (2020b). “Barley landraces: Ecological heritage for edaphic stress adaptations and sustainable production.” Environ. Sustainability Indic. 6, 100035. doi: 10.1016/j.indic.2020.100035

[B64] Lechner E. Achard P. Vansiri A. Potuschak T. Genschik P. (2006). F-box proteins everywhere. Curr. Opin. Plant Biol. 9, 631–638. doi: 10.1016/j.pbi.2006.09.003 17005440

[B65] Lee D.-K. Chung P. J. Jeong J. S. Jang G. Bang S. W. Jung H.. (2017). The rice OsNAC6 transcription factor orchestrates multiple molecular mechanisms involving root structural adaptions and nicotianamine biosynthesis for drought tolerance. Plant Biotechnol. J. 15, 754–764. doi: 10.1111/pbi.12673 27892643 PMC5425393

[B66] Leipe D. D. Koonin E. V. Aravind L. (2004). STAND, a class of p-loop NTPases including animal and plant regulators of programmed cell death: Multiple, complex domain architectures, unusual phyletic patterns, and evolution by horizontal gene transfer. J. Mol. Biol. 343, 1–28. doi: 10.1016/j.jmb.2004.08.023 15381417

[B67] Li K.-L. Bai X. Li Y. Cai H. Ji W. Tang L.-L.. (2011). GsGASA1 mediated root growth inhibition in response to chronic cold stress is marked by the accumulation of DELLAs. J. Plant Physiol. 168, 2153–2160. doi: 10.1016/j.jplph.2011.07.006 21855169

[B68] Lipka A. E. Tian F. Wang Q. Peiffer J. Li M. Bradbury P. J.. (2012). GAPIT: Genome association and prediction integrated tool. Bioinformatics 28, 2397–2399. doi: 10.1093/bioinformatics/bts444 22796960

[B69] Liu X. Huang M. Fan B. Buckler E. S. Zhang Z. (2016). Iterative usage of fixed and random effect models for powerful and efficient genome-wide association studies. PloS Genet. 12, e1005767. doi: 10.1371/journal.pgen.1005767 26828793 PMC4734661

[B70] Lohse M. Nagel A. Herter T. May P. Schroda M. Zrenner R.. (2014). Mercator: a fast and simple web server for genome scale functional annotation of plant sequence data: Mercator: sequence functional annotation server. Plant Cell Environ. 37, 1250–1258. doi: 10.1111/pce.12231 24237261

[B71] Lynch J. P. (2013). Steep, cheap and deep: An ideotype to optimize water and n acquisition by maize root systems. Ann. Bot. 112, 347–357. doi: 10.1093/aob/mcs293 23328767 PMC3698384

[B72] Lynch J. P. (2015). Root phenes that reduce the metabolic costs of soil exploration: Opportunities for 21st century agriculture. Plant Cell Environ. 38, 1775–1784. doi: 10.1111/pce.12451 25255708

[B73] Lynch J. P. Brown K. M. (2012). New roots for agriculture: Exploiting the root phenome. Philos. Trans. R. Soc. B: Biol. Sci. 367, 1598–1604. doi: 10.1098/rstb.2011.0243 PMC3321693 22527403

[B74] Lynch J. P. Chimungu J. G. Brown K. M. (2014). Root anatomical phenes associated with water acquisition from drying soil: Targets for crop improvement. J. Exp. Bot. 65, 6155–6166. doi: 10.1093/jxb/eru162 24759880

[B75] Malefo M. B. Mathibela E. O. Crampton B. G. Makgopa M. E. (2020). Investigating the role of bowman-birk serine protease inhibitor in arabidopsis plants under drought stress. Plant Physiol. Biochem. 149, 286–293. doi: 10.1016/j.plaphy.2020.02.007 32097847

[B76] Malik P. L. Janss L. Nielsen L. K. Borum F. Jørgensen H. Eriksen B.. (2019). Breeding for dual-purpose wheat varieties using marker–trait associations for biomass yield and quality traits. Theor. Appl. Genet. 132, 3375–3398. doi: 10.1007/s00122-019-03431-z 31555887

[B77] Marroni F. Pinosio S. Zaina G. Fogolari F. Felice N. Cattonaro F.. (2011). Nucleotide diversity and linkage disequilibrium in *Populus nigra* cinnamyl alcohol dehydrogenase (CAD4) gene. Tree Genet. Genomes 7, 1011–1023. doi: 10.1007/s11295-011-0391-5

[B78] Mascher M. Wicker T. Jenkins J. Plott C. Lux T. Koh C. S.. (2021). Long-read sequence assembly: a technical evaluation in barley. Plant Cell 33 (6), 1888–1906. doi: 10.1093/plcell/koab077 33710295 PMC8290290

[B79] Meyer R. C. Weigelt-Fischer K. Knoch D. Heuermann M. Zhao Y. Altmann T. (2021). Temporal dynamics of QTL effects on vegetative growth in *Arabidopsis thaliana* . J. Exp. Bot. 72, 476–490. doi: 10.1093/jxb/eraa490 33080013

[B80] Mishra M. Mahajan N. Tamhane V. A. Kulkarni M. J. Baldwin I. T. Gupta V. S.. (2012). Stress inducible proteinase inhibitor diversity in capsicum annuum. BMC Plant Biol. 12, 217. doi: 10.1186/1471-2229-12-217 23153298 PMC3511207

[B81] Miyazawa Y. Moriwaki T. Uchida M. Kobayashi A. Fujii N. Takahashi H. (2012). Overexpression of MIZU-KUSSEI1 enhances the root hydrotropic response by retaining cell viability under hydrostimulated conditions in arabidopsis thaliana. Plant Cell Physiol. 53, 1926–1933. doi: 10.1093/pcp/pcs129 23012350

[B82] Mohanan M. V. Pushpanathan A. Sasikumar S. P.T. Selvarajan D. Jayanarayanan A. N. R A. K.. (2020). Ectopic expression of DJ-1/PfpI domain containing Erianthus arundinaceus Glyoxalase III (EaGly III) enhances drought tolerance in sugarcane. Plant Cell Reports 39 (11), 1581–1594. doi: 10.1007/s00299-020-02585-1 32876807

[B83] Niu Z. Li G. Hu H. Lv J. Zheng Q. Liu J.. (2021). A gene that underwent adaptive evolution, LAC2 (LACCASE), in populus euphratica improves drought tolerance by improving water transport capacity. Hortic. Res. 8, 88. doi: 10.1038/s41438-021-00518-x 33795664 PMC8016922

[B84] Oyiga B. C. Ogbonnaya F. C. Sharma R. C. Baum M. Léon J. Ballvora A. (2019). Genetic and transcriptional variations in NRAMP-2 and OPAQUE1 genes are associated with salt stress response in wheat. Theor. Appl. Genet. 132, 323–346. doi: 10.1007/s00122-018-3220-5 30392081 PMC6349800

[B85] Oyiga B. C. Palczak J. Wojciechowski T. Lynch J. P. Naz A. A. Léon J.. (2020). Genetic components of root architecture and anatomy adjustments to water-deficit stress in spring barley. Plant Cell Environ. 43, 692–711. doi: 10.1111/pce.13683 31734943

[B86] Oyiga B. C. Sharma R. C. Baum M. Ogbonnaya F. C. Léon J. Ballvora A. (2018). Allelic variations and differential expressions detected at quantitative trait loci for salt stress tolerance in wheat. Plant Cell Environ. 41, 919–935. doi: 10.1111/pce.12898 28044314

[B87] Pacurar D. I. Pacurar M. L. Lakehal A. Pacurar A. M. Ranjan A. Bellini C. (2017). The arabidopsis Cop9 signalosome subunit 4 (CSN4) is involved in adventitious root formation. Sci. Rep. 7, 628. doi: 10.1038/s41598-017-00744-1 28377589 PMC5429640

[B88] Paez-Garcia A. Motes C. M. Scheible W. R. Chen R. Blancaflor E. B. Monteros M. J. (2015). Root traits and phenotyping strategies for plant improvement. Plants 4, 334–355. doi: 10.3390/plants4020334 27135332 PMC4844329

[B89] Palta J. A. Yang J. (2014). Crop root system behaviour and yield. Field Crops Res. 165, 1–4. doi: 10.1016/j.fcr.2014.06.024

[B90] Paradis E. Claude J. Strimmer K. (2004). APE: Analyses of phylogenetics and evolution in r language. Bioinformatics 20, 289–290. doi: 10.1093/bioinformatics/btg412 14734327

[B91] Paradis E. Schliep K. (2019). Ape 5.0: an environment for modern phylogenetics and evolutionary analyses in r. Bioinformatics 35, 526–528. doi: 10.1093/bioinformatics/bty633 30016406

[B92] Pasam R. K. Sharma R. Walther A. Özkan H. Graner A. Kilian B. (2014). Genetic diversity and population structure in a legacy collection of spring barley landraces adapted to a wide range of climates. PLoS ONE 9 (1), 129. doi: 10.1371/journal.pone.0116164 PMC4277474 25541702

[B93] Pérez-Ramos I. M. Volaire F. Fattet M. Blanchard A. Roumet C. (2013). Tradeoffs between functional strategies for resource-use and drought-survival in Mediterranean rangeland species. Environ. Exp. Bot. 87, 126–136. doi: 10.1016/j.envexpbot.2012.09.004

[B94] Pospíšilová H. Jiskrová E. Vojta P. Mrízová K. Kokáš F. Čudejková M. M.. (2016). “Transgenic barley overexpressing a cytokinin dehydrogenase gene shows greater tolerance to drought stress,” in: New biotechnology. 10.1016/j.nbt.2015.12.005 26773738

[B95] Pourkheirandish M. Komatsuda T. (2007). The importance of barley genetics and domestication in a global perspective. Ann. Bot. 100, 999–1008. doi: 10.1093/aob/mcm139 17761690 PMC2759206

[B96] Pritchard J. K. Stephens M. Donnelly P. (2000). Inference of population structure using multilocus genotype data. Genetics 155, 945–959. doi: 10.1093/genetics/155.2.945 10835412 PMC1461096

[B97] R Core Team. (2021). R: A language and environment for statistical computing. Vienna, Austria: R Foundation for statistical Computing. Available at: https://www.R-project.org/.

[B98] Remington D. L. Thornsberry J. M. Matsuoka Y. Wilson L. M. Whitt S. R. Doebley J.. (2001). Structure of linkage disequilibrium and phenotypic associations in the maize genome. Proc. Natl. Acad. Sci. U.S.A. 98, 11479–11484. doi: 10.1073/pnas.201394398 11562485 PMC58755

[B99] Richard C. Hickey L. T. Fletcher S. Jennings R. Chenu K. Christopher J. T. (2015). High-throughput phenotyping of seminal root traits in wheat. Plant Methods 11, 13. doi: 10.1186/s13007-015-0055-9 25750658 PMC4351910

[B100] Riedelsheimer C. Lisec J. Czedik-Eysenberg A. Sulpice R. Flis A. Grieder C.. (2012). Genome-wide association mapping of leaf metabolic profiles for dissecting complex traits in maize. Proc. Natl. Acad. Sci. 109, 8872–8877. doi: 10.1073/pnas.1120813109 22615396 PMC3384205

[B101] Robinson H. Hickey L. Richard C. Mace E. Kelly A. Borrell A.. (2016). Genomic regions influencing seminal root traits in barley. Plant Genome 9. doi: 10.3835/plantgenome2015.03.0012 27898766

[B102] Sachdeva S. Bharadwaj C. Singh R. K. Jain P. K. Patil B. S. Roorkiwal M.. (2020). Characterization of ASR gene and its role in drought tolerance in chickpea (Cicer arietinum l.). PloS One 15, e0234550. doi: 10.1371/journal.pone.0234550 32663226 PMC7360048

[B103] Saisho D. Takeda K. (2011). Barley: Emergence as a new research material of crop science. Plant Cell Physiol. 52, 724–727. doi: 10.1093/pcp/pcr049 21565909

[B104] Saitou N. Nei M. (1987). The neighbor-joining method: A new method for reconstructing phylogenetic trees. Mol Biol Evol 4 (4), 406–425. doi: 10.1093/oxfordjournals.molbev.a040454 3447015

[B105] Sallam A. Alqudah A. M. Dawood M. F. A. Baenziger P. S. Börner A. (2019). Drought stress tolerance in wheat and barley: Advances in physiology, breeding and genetics research. Int. J. Mol. Sci. 20 (13), 3137. doi: 10.3390/ijms20133137 31252573 PMC6651786

[B106] Schonfeld M. A. Johnson R. C. Carver B. F. Mornhinweg D. W. (1988). Water relations in winter wheat as drought resistance indicators. Crop Sci. 28, 526–531. doi: 10.2135/cropsci1988.0011183X002800030021x

[B107] Schwacke R. Ponce-Soto G. Y. Krause K. Bolger A. M. Arsova B. Hallab A.. (2019). MapMan4: A refined protein classification and annotation framework applicable to multi-omics data analysis. Mol. Plant 12, 879–892. doi: 10.1016/j.molp.2019.01.003 30639314

[B108] Sevilla F. Camejo D. Ortiz-Espín A. Calderón A. Lázaro J. J. Jiménez A. (2015). The thioredoxin/peroxiredoxin/sulfiredoxin system: current overview on its redox function in plants and regulation by reactive oxygen and nitrogen species. J. Exp. Bot. 66, 2945–2955. doi: 10.1093/jxb/erv146 25873657

[B109] Shan L. E. I. Li C. Chen F. Zhao S. Xia G. (2008). A bowman-birk type protease inhibitor is involved in the tolerance to salt stress in wheat. Plant Cell Environ. 31, 1128–1137. doi: 10.1111/j.1365-3040.2008.01825.x 18433440

[B110] Sharma N. K. Gupta S. K. Dwivedi V. Chattopadhyay D. (2020). Lignin deposition in chickpea root xylem under drought. Plant Signaling Behav. 15, 1754621. doi: 10.1080/15592324.2020.1754621 PMC8570712 32290771

[B111] Sharma M. Singh A. Shankar A. Pandey A. Baranwal V. Kapoor S.. (2014). Comprehensive expression analysis of rice armadillo gene family during abiotic stress and development. DNA Res. 21, 267–283. doi: 10.1093/dnares/dst056 24398598 PMC4060948

[B112] Shi H. Chen L. Ye T. Liu X. Ding K. Chan Z. (2014). Modulation of auxin content in arabidopsis confers improved drought stress resistance. Plant Physiol. Biochem. 82, 209–217. doi: 10.1016/j.plaphy.2014.06.008 24992887

[B113] Shin J.-H. Blay S. Graham J. McNeney B. (2006). LDheatmap: An r function for graphical display of pairwise linkage disequilibria between single nucleotide polymorphisms. J. Stat. Software 16 (3), 1–9. doi: 10.18637/jss.v016.c03

[B114] Singh A. K. Chamovitz D. A. (2019). Role of Cop9 signalosome subunits in the environmental and hormonal balance of plant. Biomolecules 9 (6), 224. doi: 10.3390/biom9060224 31181827 PMC6628103

[B115] Srinivasan T. Kumar K. R. R. Kirti P. B. (2009). Constitutive expression of a trypsin protease inhibitor confers multiple stress tolerance in transgenic tobacco. Plant Cell Physiol. 50, 541–553. doi: 10.1093/pcp/pcp014 19179349

[B116] Stacklies W. Redestig H. Scholz M. Walther D. Selbig J. (2007). pcaMethods–a bioconductor package providing PCA methods for incomplete data. Bioinformatics 23, 1164–1167. doi: 10.1093/bioinformatics/btm069 17344241

[B117] Steffens B. Rasmussen A. (2016). The physiology of adventitious roots. Plant Physiol. 170, 603–617. doi: 10.1104/pp.15.01360 26697895 PMC4734560

[B118] Stich B. Melchinger A. E. (2009). Comparison of mixed-model approaches for association mapping in rapeseed, potato, sugar beet, maize, and arabidopsis. BMC Genomics 10, 94. doi: 10.1186/1471-2164-10-94 19250529 PMC2676307

[B119] Stich B. Möhring J. Piepho H.-P. Heckenberger M. Buckler E. S. Melchinger A. E. (2008). Comparison of mixed-model approaches for association mapping. Genetics 178, 1745–1754. doi: 10.1534/genetics.107.079707 18245847 PMC2278052

[B120] Sullivan P. Arendt E. Gallagher E. (2013). The increasing use of barley and barley by-products in the production of healthier baked goods. Trends Food Sci. Technol. 29, 124–134. doi: 10.1016/j.tifs.2012.10.005

[B121] Sun H. Tao J. Gu P. Xu G. Zhang Y. (2016). The role of strigolactones in root development. Plant Signal Behav. 11, e1110662–e1110662. doi: 10.1080/15592324.2015.1110662 26515106 PMC4871655

[B122] Szkopińska A. Płochocka D. (2005). Farnesyl diphosphate synthase; regulation of product specificity. Acta Biochim. Pol. 52, 45–55. doi: 10.18388/abp.2005_3485 15827605

[B123] Tagliotti M. E. Deperi S. I. Bedogni M. C. Huarte M. (2021). Genom … wide association analysis of agronomical and physiological traits linked to drought tolerance in a diverse potatoes (Solanum tuberosum) panel. Plant Breeding. 140, 654–664. doi: 10.1111/pbr.12938

[B124] Tombuloglu H. (2019). Genome-wide analysis of the auxin response factors (ARF) gene family in barley (Hordeum vulgare l.). J. Plant Biochem. Biotechnol. 28, 14–24. doi: 10.1007/s13562-018-0458-6

[B125] Turner A. Beales J. Faure S. Dunford R. P. Laurie D. A. (2005). The pseudo-response regulator ppd-H1 provides adaptation to photoperiod in barley. Science 310, 1031–1034. doi: 10.1126/science.1117619 16284181

[B126] Vadez V. Rao J. S. Bhatnagar-Mathur P. Sharma K. K. (2013). *DREB1A* promotes root development in deep soil layers and increases water extraction under water stress in groundnut: *DREB1A* promotes root development in groundnut. Plant Biol. 15, 45–52. doi: 10.1111/j.1438-8677.2012.00588.x 22672619

[B127] VanRaden P. M. (2008). Efficient methods to compute genomic predictions. J. Dairy Sci. 91, 4414–4423. doi: 10.3168/jds.2007-0980 18946147

[B128] Vaseva I. I. Zehirov G. Kirova E. Simova-Stoilova L. (2016). Transcript profiling of serine- and cysteine protease inhibitors inTriticum aestivumvarieties with different drought tolerance. Cereal Res. Commun. 44, 79–88. doi: 10.1556/0806.43.2015.032

[B129] Verslues P. E. Lasky J. R. Juenger T. E. Liu T.-W. Kumar M. N. (2014). Genome-wide association mapping combined with reverse genetics identifies new effectors of low water potential-induced proline accumulation in arabidopsis. Plant Physiol. 164, 144–159. doi: 10.1104/pp.113.224014 24218491 PMC3875797

[B130] Vessal S. Arefian M. Siddique K. H. M. (2020). Proteomic responses to progressive dehydration stress in leaves of chickpea seedlings. BMC Genomics 21 (1), 523. doi: 10.1186/s12864-020-06930-2 32727351 PMC7392671

[B131] Virlouvet L. Jacquemot M. P. Gerentes D. Corti H. Bouton S. Gilard F.. (2011). The ZmASR1 protein influences branched-chain amino acid biosynthesis and maintains kernel yield in maize under water-limited conditions. Plant Physiol. 157, 917–936. doi: 10.1104/pp.111.176818 21852416 PMC3192578

[B132] Vlamis J. Williams D. E. (1962). Ion competition in manganese uptake by barley plants. Plant Physiol. 37, 650–655. doi: 10.1104/pp.37.5.650 16655709 PMC549850

[B133] Voorrips R. E. (2002). MapChart: Software for the graphical presentation of linkage maps and QTLs. J. Heredity 93, 77–78. doi: 10.1093/jhered/93.1.77 12011185

[B134] Wabila C. Neumann K. Kilian B. Radchuk V. Graner A. (2019). A tiered approach to genome-wide association analysis for the adherence of hulls to the caryopsis of barley seeds reveals footprints of selection. BMC Plant Biol. 19, 95. doi: 10.1186/s12870-019-1694-1 30841851 PMC6404267

[B135] Waititu J. K. Zhang X. Chen T. Zhang C. Zhao Y. Wang H. (2021). Transcriptome analysis of tolerant and susceptible maize genotypes reveals novel insights about the molecular mechanisms underlying drought responses in leaves. IJMS 22 (13), 6980. doi: 10.3390/ijms22136980 34209553 PMC8268334

[B136] Wang G. Cai G. Xu N. Zhang L. Sun X. Guan J.. (2019). Novel DnaJ protein facilitates thermotolerance of transgenic tomatoes. IJMS 20, 367. doi: 10.3390/ijms20020367 30654548 PMC6359579

[B137] Wang X.-F. He F.-F. Ma X.-X. Mao C.-Z. Hodgman C. Lu C.-G.. (2011). OsCAND1 is required for crown root emergence in rice. Mol. Plant 4, 289–299. doi: 10.1093/mp/ssq068 20978084

[B138] Wang Y. Wang M. Sun Y. Wang Y. Li T. Chai G.. (2015). FAR5, a fatty acyl-coenzyme a reductase, is involved in primary alcohol biosynthesis of the leaf blade cuticular wax in wheat (Triticum aestivum l.). J. Exp. Bot. 66, 1165–1178. doi: 10.1093/jxb/eru457 25468933 PMC4438443

[B139] Wang Y. Xu J. He Z. Hu N. Luo W. Liu X.. (2021). BdFAR4, a root-specific fatty acyl-coenzyme a reductase, is involved in fatty alcohol synthesis of root suberin polyester in *Brachypodium distachyon* . Plant J. 106, 1468–1483. doi: 10.1111/tpj.15249 33768632

[B140] Wang Y. Ying J. Zhang Y. Xu L. Zhang W. Ni M.. (2020). Genome-wide identification and functional characterization of the cation proton antiporter (CPA) family related to salt stress response in radish (Raphanus sativus l.). Int. J. Mol. Sci. 21 (21), 8262. doi: 10.3390/ijms21218262 33158201 PMC7662821

[B141] Wang J. Zhang Z. (2021). GAPIT version 3: Boosting power and accuracy for genomic association and prediction. Genom. Proteom. Bioinform. 19 (4), 629–640. doi: 10.1016/j.gpb.2021.08.005 PMC9121400 34492338

[B142] Wasson A. P. Richards R. A. Chatrath R. Misra S. C. Prasad S. V. S. Rebetzke G. J.. (2012). Traits and selection strategies to improve root systems and water uptake in water-limited wheat crops. J. Exp. Bot. 63, 3485–3498. doi: 10.1093/jxb/ers111 22553286

[B143] Wehner G. G. Balko C. C. Enders M. M. Humbeck K. K. Ordon F. F. (2015). Identification of genomic regions involved in tolerance to drought stress and drought stress induced leaf senescence in juvenile barley. BMC Plant Biol. 15, 125. doi: 10.1186/s12870-015-0524-3 25998066 PMC4440603

[B144] Wong M. M. Bhaskara G. B. Wen T.-N. Lin W.-D. Nguyen T. T. Chong G. L.. (2019). Phosphoproteomics of arabidopsis highly ABA-Induced1 identifies AT-Hook-Like10 phosphorylation required for stress growth regulation. Proc. Natl. Acad. Sci. U.S.A. 116, 2354–2363. doi: 10.1073/pnas.1819971116 30670655 PMC6369736

[B145] Würschum T. Tucker M. R. Reif J. C. Maurer H. (2012). Improved efficiency of doubled haploid generation in hexaploid triticale by *in vitro* chromosome doubling. BMC Plant Biol. 12, 109. doi: 10.1186/1471-2229-12-109 22809089 PMC3443072

[B146] Xia Z. Zhang X. Li J. Su X. Liu J. (2014). Overexpression of a tobacco J-domain protein enhances drought tolerance in transgenic arabidopsis. Plant Physiol. Biochem. 83, 100–106. doi: 10.1016/j.plaphy.2014.07.023 25128645

[B147] Xie Q. Frugis G. Colgan D. Chua N. H. (2000). Arabidopsis NAC1 transduces auxin signal downstream of TIR1 to promote lateral root development. Genes Dev. 14, 3024–3036. doi: 10.1101/gad.852200 11114891 PMC317103

[B148] Xu X. M. Lin H. Maple J. Björkblom B. Alves G. Larsen J. P.. (2010). The *Arabidopsis* DJ-1a protein confers stress protection through cytosolic SOD activation. J. Cell Sci. 123, 1644–1651. doi: 10.1242/jcs.063222 20406884

[B149] Yang L. Zheng B. Mao C. Qi X. Liu F. Wu P. (2004). Analysis of transcripts that are differentially expressed in three sectors of the rice root system under water deficit. Mol. Genet. Genomics 272, 433–442. doi: 10.1007/s00438-004-1066-9 15480789

[B150] Yin L. Zhang H. Tang Z. Xu J. Yin D. Zhang Z.. (2021). rMVP: A memory-efficient, visualization-enhanced, and parallel-accelerated tool for genome-wide association study. Genomics Proteomics Bioinf. 19, 619–628. doi: 10.1016/j.gpb.2020.10.007 PMC9040015 33662620

[B151] Zadražnik T. Moen A. Egge-Jacobsen W. Meglič V. Šuštar-Vozlič J. (2017). Towards a better understanding of protein changes in common bean under drought: A case study of n-glycoproteins. Plant Physiol. biochemistry: PPB 118, 400–412. doi: 10.1016/j.plaphy.2017.07.004 28711789

[B152] Žárský V. Kulich I. Fendrych M. Pečenková T. (2013). Exocyst complexes multiple functions in plant cells secretory pathways. Curr. Opin. Plant Biol. 16, 726–733. doi: 10.1016/j.pbi.2013.10.013 24246229

[B153] Zhang Q. Liu H. Wu X. Wang W. (2020). Identification of drought tolerant mechanisms in a drought-tolerant maize mutant based on physiological, biochemical and transcriptomic analyses. BMC Plant Biol. 20, 315. doi: 10.1186/s12870-020-02526-w 32620139 PMC7350183

[B154] Zhao Y. Cheng X. Liu X. Wu H. Bi H. Xu H. (2018). The wheat MYB transcription factor TaMYB(31) is involved in drought stress responses in arabidopsis. Front. Plant Sci. 9. doi: 10.3389/fpls.2018.01426 PMC6172359 30323824

[B155] Zhao Q. Nakashima J. Chen F. Yin Y. Fu C. Yun J.. (2013). *LACCASE* is necessary and nonredundant with *PEROXIDASE* for lignin polymerization during vascular development in *Arabidopsis* . Plant Cell 25, 3976–3987. doi: 10.1105/tpc.113.117770 24143805 PMC3877815

[B156] Zhou Z. Li Q. Xiao L. Wang Y. Feng J. Bu Q.. (2021). Multiplexed CRISPR/Cas9-mediated knockout of laccase genes in salvia miltiorrhiza revealed their roles in growth, development, and metabolism. Front. Plant Sci. 12. doi: 10.3389/fpls.2021.647768 PMC8014014 33815454

[B157] Zhou H. Qi K. Liu X. Yin H. Wang P. Chen J.. (2016). Genome-wide identification and comparative analysis of the cation proton antiporters family in pear and four other rosaceae species. Mol. Genet. Genomics 291, 1727–1742. doi: 10.1007/s00438-016-1215-y 27193473

[B158] Zimmermann R. Sakai H. Hochholdinger F. (2009). The *Gibberellic acid stimulated-like* gene family in maize and its role in lateral root development. Plant Physiol. 152, 356–365. doi: 10.1104/pp.109.149054 19926801 PMC2799369

