# Supplementary material for: Genome-wide association study in two-row spring barley landraces identifies QTL associated with plantlets root system architecture traits in well-watered and osmotic stress conditions
Source: Front Plant Sci. 2023 Apr 3;14:1125672. doi: 10.3389/fpls.2023.1125672 (PMC10106628; doi:10.3389/fpls.2023.1125672)
Supplement: Supplementary file 2 [file Image_1.pdf]

Supplementary Material

Genome-wide association study in two-row spring barley landraces identifies QTLs associated with plantlets root system architecture traits in well-watered and osmotic stress conditions

Mortaza Khodaeiaminjan[1*], Dominic Knoch[2], Marie Rose Ndella Thiaw[3], Cintia Marchetti[1], Nikola Kořínková[1], Alexie Techer[1], Thu D. Nguyen[1], Jianting Chu[4], Valentin Bertholomey[5], Ingrid Doridant[5], Pascal Gantet[1,3], Andreas Graner[6], Kerstin Neumann[2], Véronique Bergougnoux[1*]

*** Correspondence:** Véronique Bergougnoux: veronique.bergougnoux@upol.cz; Mortaza Khodaeiaminjan : mortaza.khodaeiaminjan@ucd.ie

1 Supplementary Figures

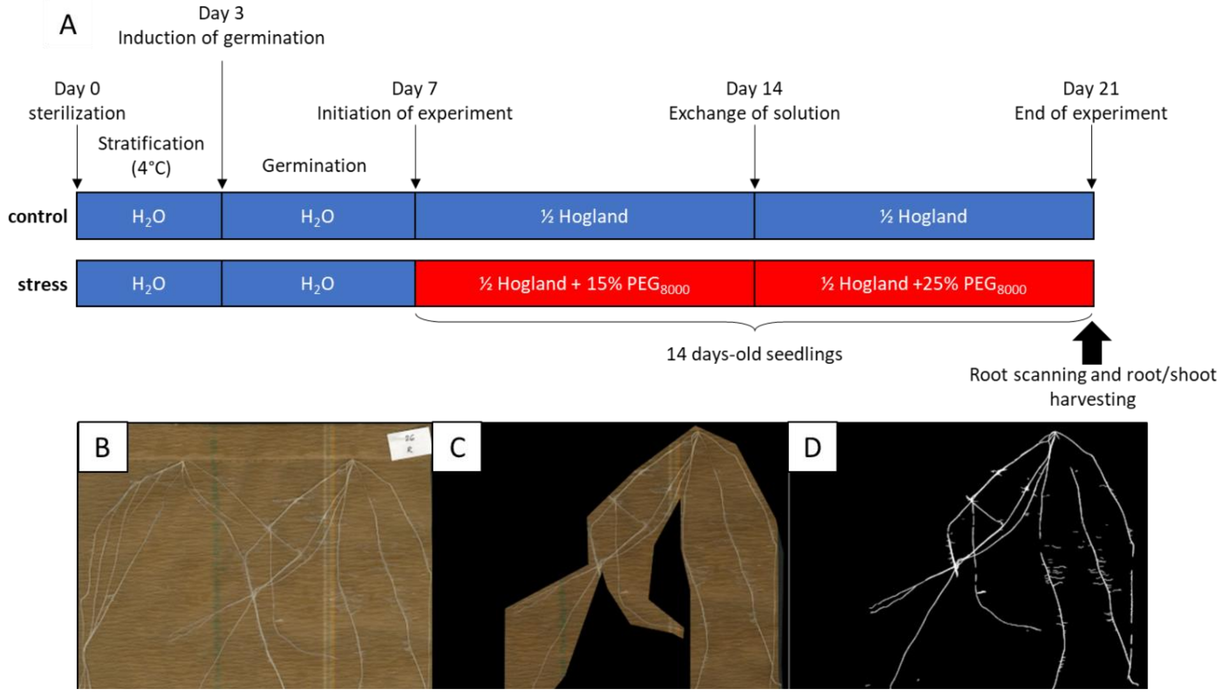

Supplemental Fig. S1. Schematic representation of the experimental design (A) and processing root images in ImageJ macro (B-D). Image of the roots (B) were processed in such way that they were first individualized by cropping (C) and converted into white pixels on black background (D) using an ImageJ macro. The converted images were analyzed with GiA Roots software to obtain RSA traits.pixels on black background (C) using an ImageJ macro. The converted images were analyzed with GiA Roots software to obtain RSA traits.

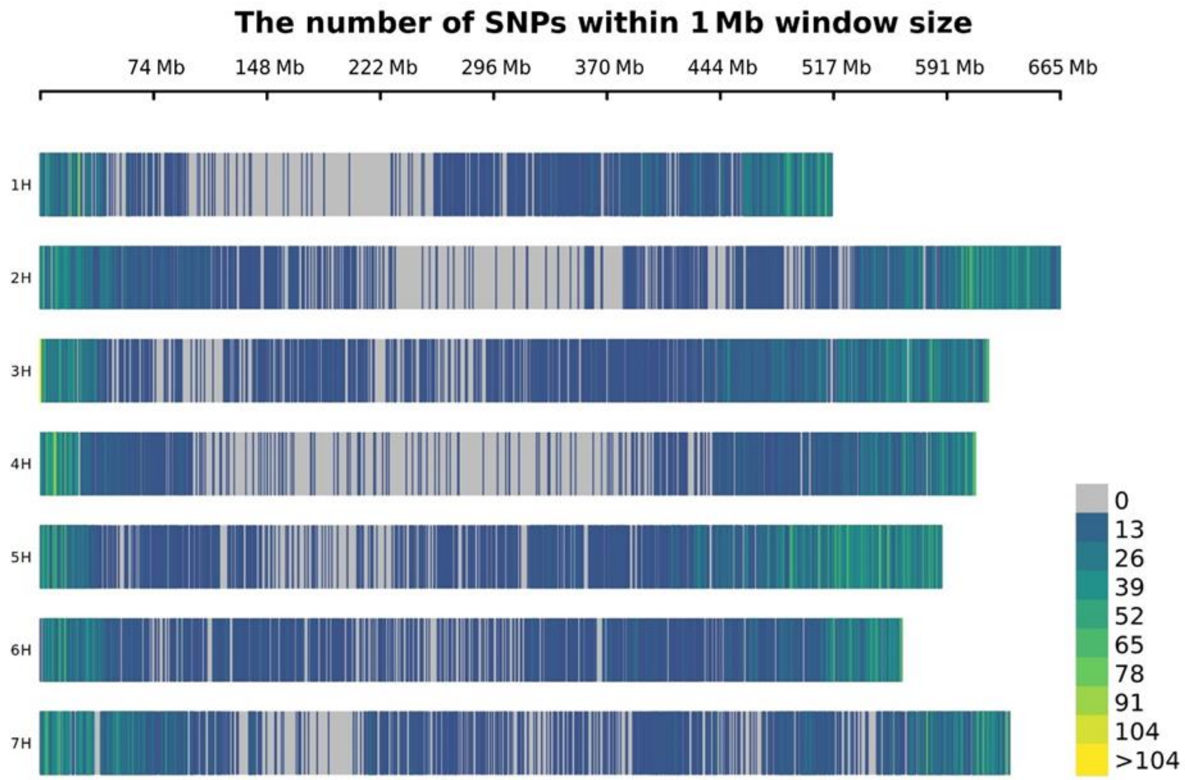

Supplementary Fig. S2. Distribution of genetic markers across the seven barley chromosomes. Shown is the genome-wide SNP marker distribution across the seven Barley chromosomes. 32,286 high-quality SNP markers were binned in 1 Mb intervals. The marker density is indicated by the colour legend (viridis colour scale) on the right side. Grey colour indicates regions without genetic markers.

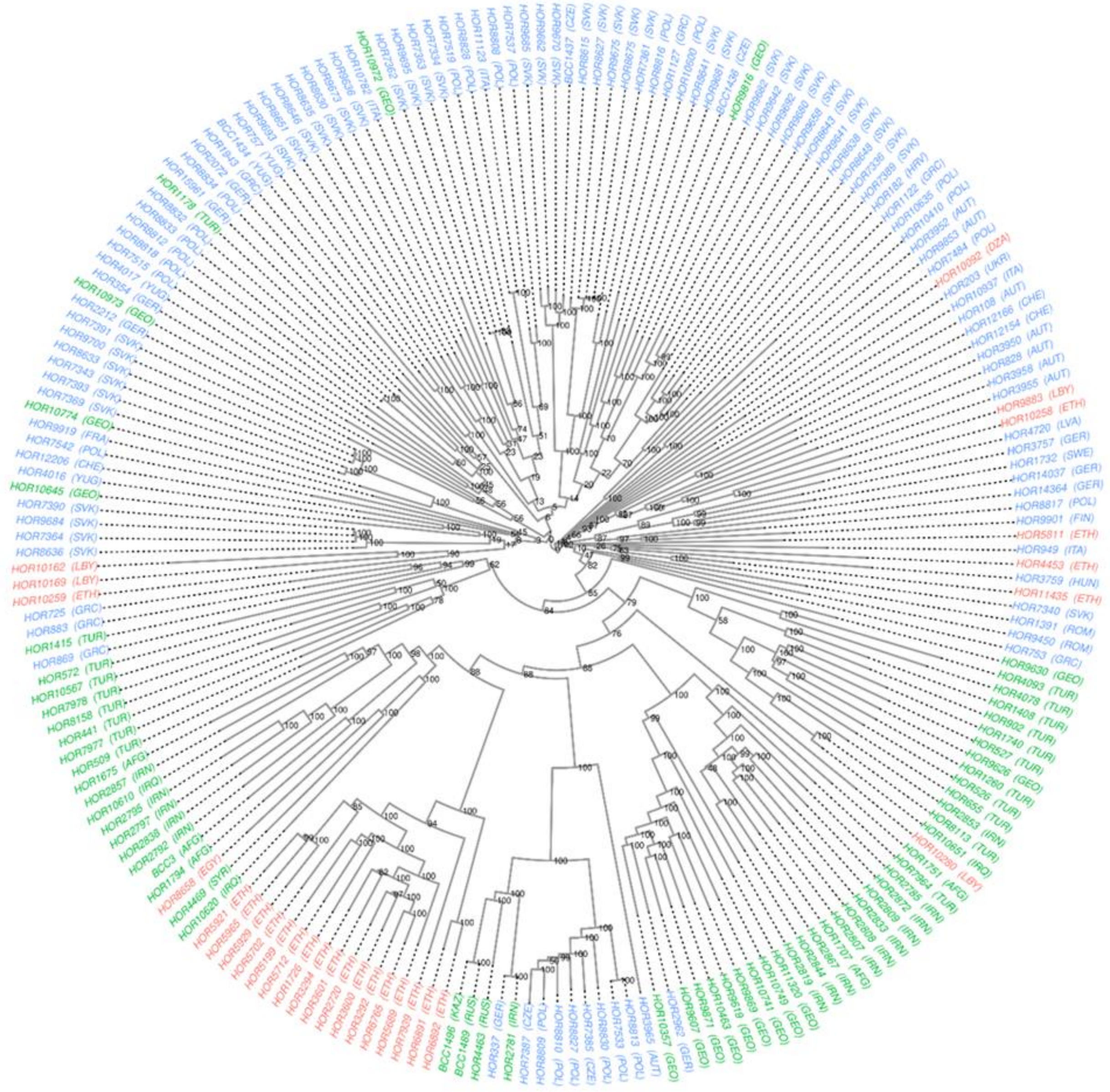

Supplementary Fig. S3. Visualisation of population structure (NJ tree). The neighbour joining tree was calculated for the panel of 206 two-rowed Barley landraces using 32,286 SNP markers to visualize population structure. Different colours correspond to the geographic origin (continent: red = Africa, green = Asia, blue= Europe) of the lines according to collection information, countries are abbreviated using a three-letter code. Bootstrap confidence levels with 100 replicates for the phylogenetic tree are indicated by the values at the nodes.

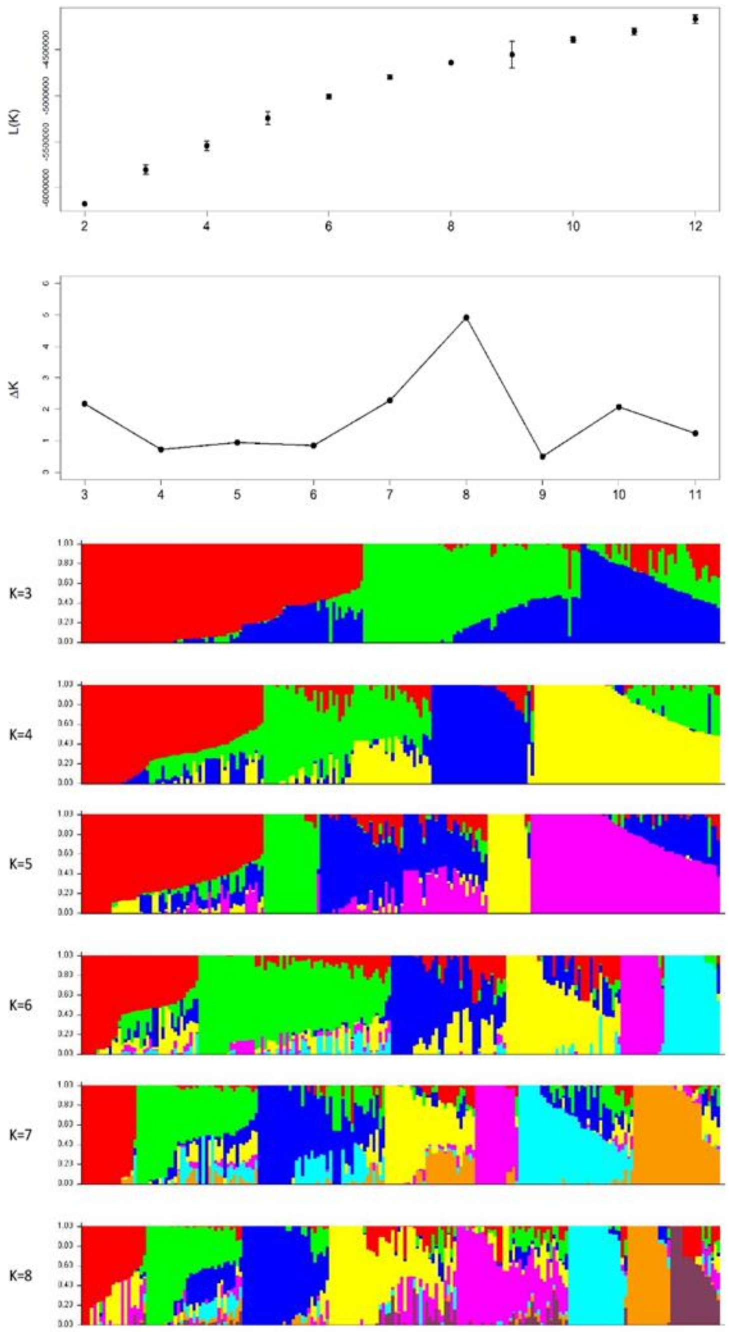

Supplementary Fig. S4. STRUCTURE analysis. Population structure for the 206 Barley lines subjected to GWAS was analysed using the programme STRUCTURE, version 2.3.4 (Pritchard *et al.*, 2000). Population clustering for K= 1 to 12 was performed using the 'admixture' model with a burn-in period of 10.000, 50.000 MCMC replications and 10 iterations per K. Mean Ln probability L(K) and standard deviation for K= 2 to 12, ΔK according to Evanno *et al*. (2005) for K= 3 to 11 and plots for K= 3 to 8. Barley lines are represented by thin vertical lines, which are partitioned into K coloured segments representing the individual's estimated membership fractions and were by their ancestry vector (Q) are given. Lines with multiple colours have admixture from multiple clusters.

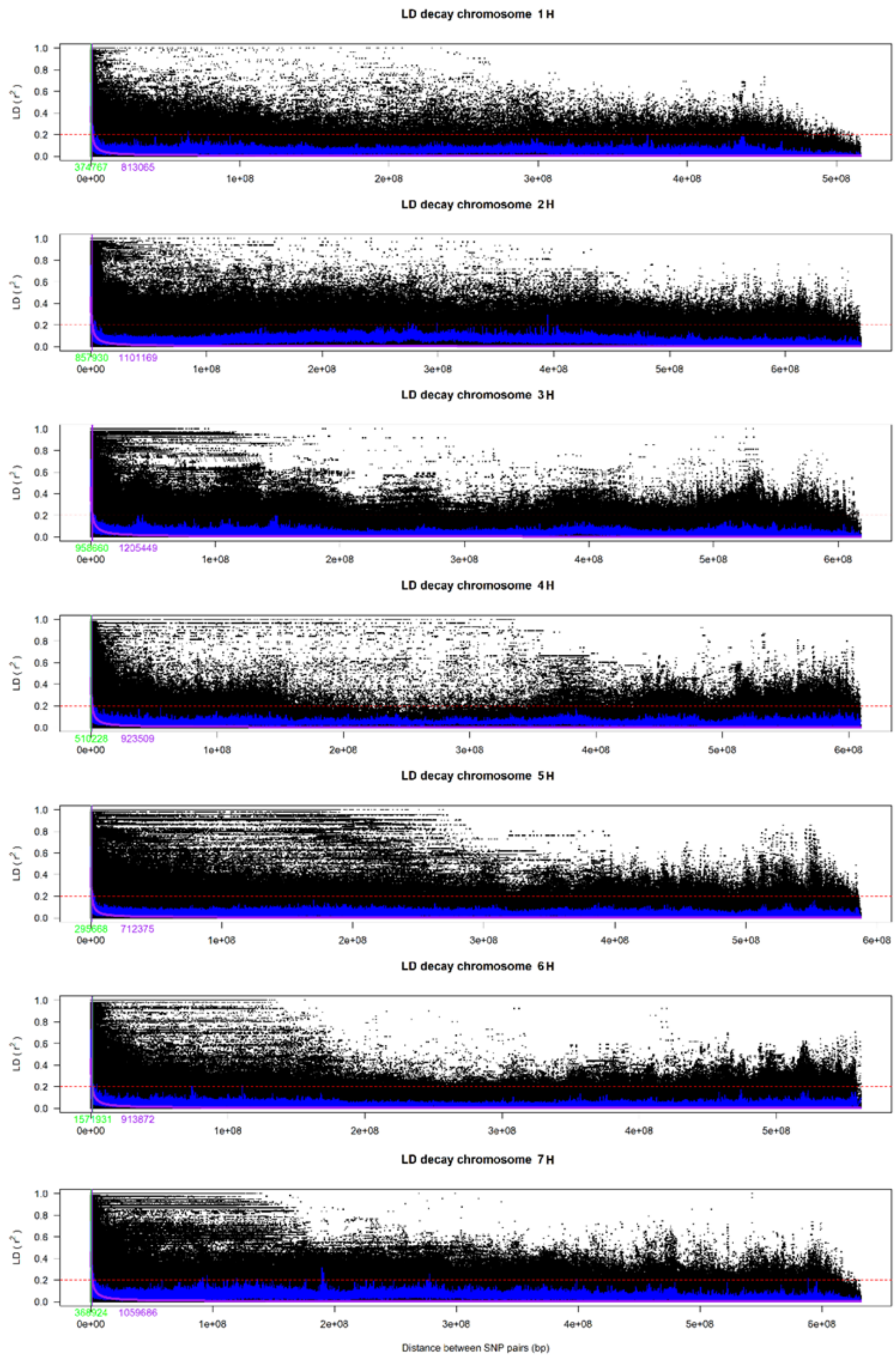

Supplementary Fig. S5. LD-decay of the seven barley chromosomes. Pairwise marker linkage disequilibrium (LD) was calculated as r^2 values from the SNP data and plotted against the physical marker distances on the Barley (Morex v3) genomes, separately for all seven chromosomes. The blue line represents a rolling mean of LD of 100 markers. The purple line shows the LD decay calculated according to Hill and Weir (1988) and modified by Remington *et al*. (2001) using a non-linear model. The horizontal dashed line corresponds to a LD value of 0.2 and the vertical

green line to the distance where the rolling mean drops below this value. The values for half-decay and LD 0.2 are given in bp below the chromosomes in purple and green color, respectively.

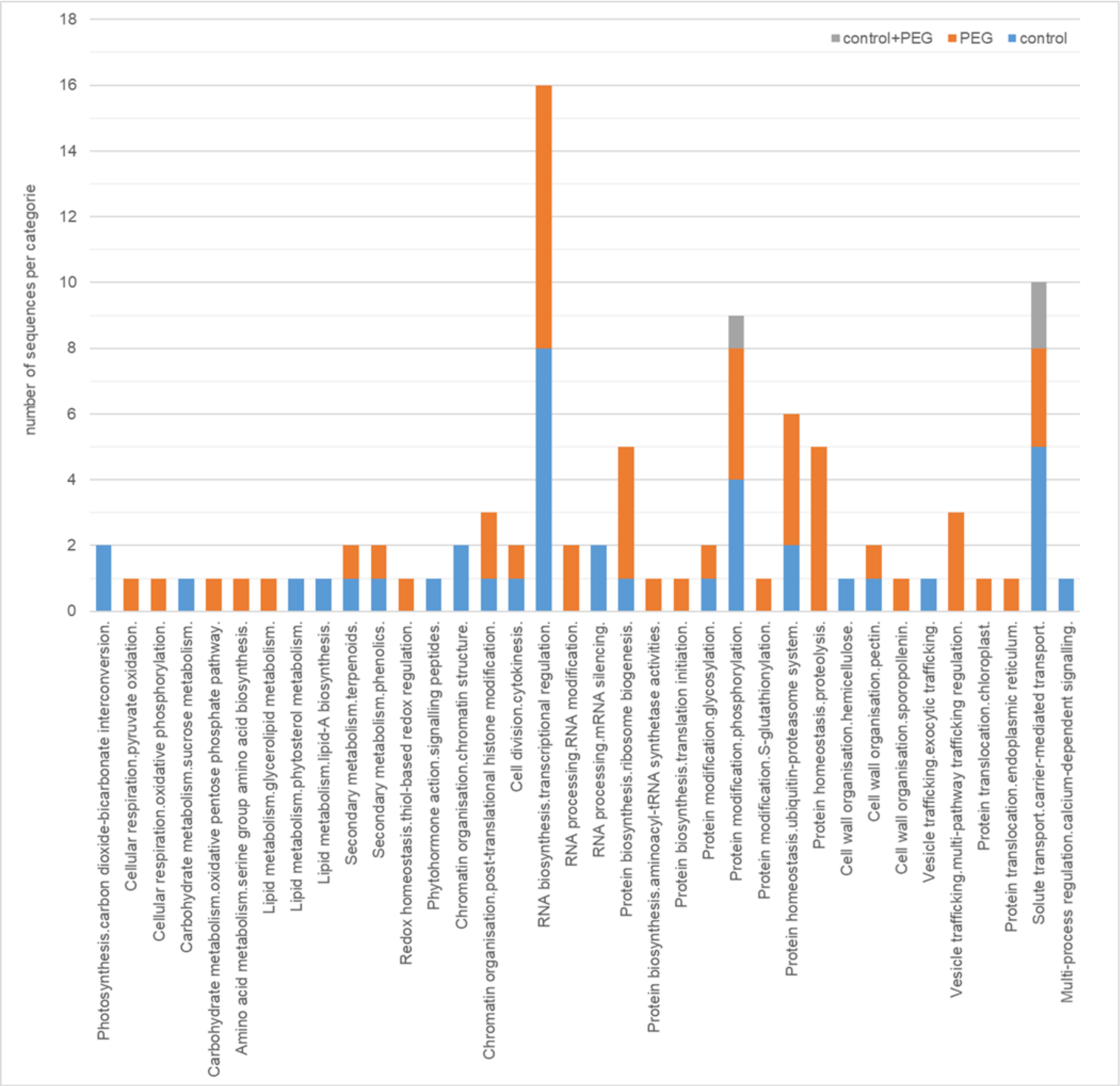

Supplementary Fig. S6. Representation of BIN functional annotation for genes associated with traits in control (blue), stress (orange) or control/stress (grey) conditions. Sequences of genes within LD-blocks were retrieved from the reference MorexV3 genome and functionally annotated with Mercator; genes entering a specific annotation at the level 2 were counted and plotted as a stack column representation of number of sequences annotated in the different BIN categories. The use of different colours ensures a fast identification of processes related to markers associated either with control or osmotic stress, or common to both treatments.
